# The genome landscape of indigenous African cattle

**DOI:** 10.1186/s13059-017-1153-y

**Published:** 2017-02-20

**Authors:** Jaemin Kim, Olivier Hanotte, Okeyo Ally Mwai, Tadelle Dessie, Salim Bashir, Boubacar Diallo, Morris Agaba, Kwondo Kim, Woori Kwak, Samsun Sung, Minseok Seo, Hyeonsoo Jeong, Taehyung Kwon, Mengistie Taye, Ki-Duk Song, Dajeong Lim, Seoae Cho, Hyun-Jeong Lee, Duhak Yoon, Sung Jong Oh, Stephen Kemp, Hak-Kyo Lee, Heebal Kim

**Affiliations:** 1 C&K genomics, Seoul National University Research Park, Seoul, 151-919 Republic of Korea; 2 The University of Nottingham, School of Life Sciences, Nottingham, NG7 2RD UK; 3 International Livestock Research institute (ILRI), P. O. Box 5689, Addis Ababa, Ethiopia; 4 International Livestock Research Institute (ILRI), Box 30709 -00100, Nairobi, Kenya; 5 Department of Parasitology, Faculty of Veterinary Medicine, University of Khartoum, 13314 Khartoum North, Sudan; 6 National Coordinateur RGA, Ministère Elevage – Productions Animales, B.P. 559, Conakry, Guinea; 7 Nelson Mandela African Institution of Science and Technology, Nelson Mandela Road. P. O. Box 447, Arusha, Tanzania; 8 Interdisciplinary Program in Bioinformatics, Seoul National University, Seoul, 151-741 Republic of Korea; 9 Department of Animal Sciences, University of Illinois, Urbana, IL 61801 USA; 10 Department of Agricultural Biotechnology and Research Institute of Agriculture and Life Sciences, Seoul National University, Seoul, 151-742 Republic of Korea; 11 College of Agriculture and Environmental Sciences, Bahir Dar University, P. O. Box 79, Bahir Dar, Ethiopia; 12 The Animal Molecular Genetics and Breeding Center, Chonbuk National University, Jeonju, 54896 Republic of Korea; 13 Division of Animal Genomics & Bioinformatics, National Institute of Animal Science, RDA, Jeonju, 565-851 Republic of Korea; 14 Animal Nutritional & Physiology Team, National Institute of Animal Science, RDA, Jeonju, 565-851 Republic of Korea; 15 Department of Animal Science, Kyungpook National University, Sangju, 742-711 Republic of Korea; 16 National Institute of Animal Science, RDA, Jeonju, 565-851 Republic of Korea; 17 The Centre for Tropical Livestock Genetics and Health, The Roslin Institute, The University of Edinburgh, Easter Bush Campus, Midlothian, EH25 9RG UK; 18 Department of Animal Biotechnology, Chonbuk National University, Jeonju, 561-756 Republic of Korea; 19 Institute for Biomedical Sciences, Shinshu University, Nagano, Japan

**Keywords:** African cattle, Genome, Adaptation, Diversity

## Abstract

**Background:**

The history of African indigenous cattle and their adaptation to environmental and human selection pressure is at the root of their remarkable diversity. Characterization of this diversity is an essential step towards understanding the genomic basis of productivity and adaptation to survival under African farming systems.

**Results:**

We analyze patterns of African cattle genetic variation by sequencing 48 genomes from five indigenous populations and comparing them to the genomes of 53 commercial taurine breeds. We find the highest genetic diversity among African zebu and sanga cattle. Our search for genomic regions under selection reveals signatures of selection for environmental adaptive traits. In particular, we identify signatures of selection including genes and/or pathways controlling anemia and feeding behavior in the trypanotolerant N’Dama, coat color and horn development in Ankole, and heat tolerance and tick resistance across African cattle especially in zebu breeds.

**Conclusions:**

Our findings unravel at the genome-wide level, the unique adaptive diversity of African cattle while emphasizing the opportunities for sustainable improvement of livestock productivity on the continent.

**Electronic supplementary material:**

The online version of this article (doi:10.1186/s13059-017-1153-y) contains supplementary material, which is available to authorized users.

## Background

Cattle are central to the African economy and society. The so-called “African Cattle Complex” refers to their role as walking larder, as a source of traction and manure, as well as to their societal importance, including during marriage, birth, death, and/or initiation ceremonies, and their representation of power, prestige, and status [1–3].

The earliest cattle of Africa were of taurine *Bos taurus* type. Subsequent waves of migrations of humped zebu *B. indicus* animals then reshaped the genomic landscape of African cattle [4–6]. Today, the African continent is uniquely rich in cattle diversity with around 150 African cattle breeds or populations recognized [7, 8]. These are grouped according to their phenotypes into taurine, zebu, and the ancient stabilized taurine × zebu crossbreed known as sanga [6]. Importantly, it is now well established that African cattle carry a taurine maternal ancestry originating from the Near East taurine domestication center(s), while the possible genetic contribution of the now extinct African auroch *B. primigenius opisthonomous* remains unclear [9, 10]. The pattern of introgression of the zebu genome across the South, East, and the North-Western part of sub-Saharan Africa has been well-documented using autosomal and *Y*-specific microsatellite loci [4–6].

**Fig. 1:**
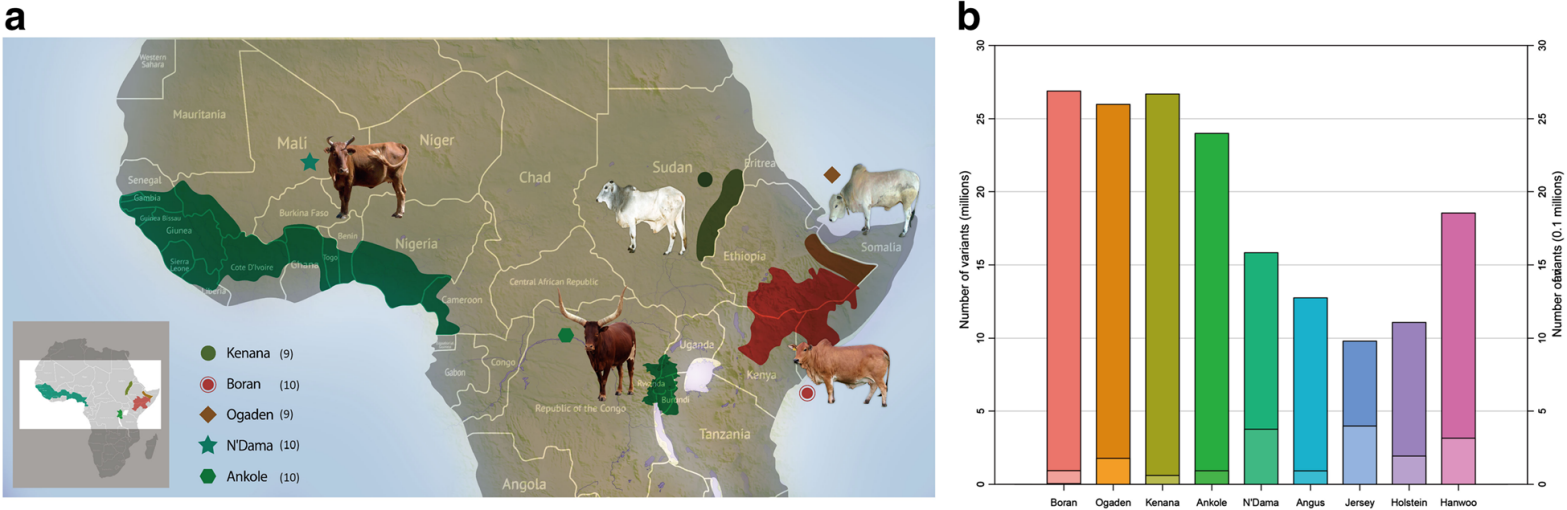
**a** Geographic locations of African cattle populations. **b** Nucleotide genome diversity. Number of single nucleotide polymorphisms (SNPs) identified in each breed (*left y-axis*) with respect to the reference genome (UMD 3.1). *Lower bars* represent the number of breed-specific SNPs (*right y-axis*)

African cattle inhabit more than five distinct agro-ecological zones [11]. Overall, zebu cattle are common in the arid and semi-arid northern Sahelo-Sudanian zone as well as on the eastern part of the continent including the highlands; whereas taurine cattle today form the majority of the herds in the sub-humid and humid regions of West Africa, which are heavily infested with the vector of African trypanosomes, the tsetse fly [11]. Sanga cattle are predominantly found in the western region of central Africa around the Great Lakes region and on the southern part of the continent [11] (Fig. 1a). African cattle populations have been subjected to strong environmental pressures including hot, dry, or humid tropical climate conditions and heavy and diverse disease challenges. Accordingly, they are expected to display unique adaptive traits. This is exemplified by the trypanotolerance traits of the N’Dama and other West African taurine breeds inhabiting the tsetse-infested areas [12]. African cattle have also been shaped by human selection for traits such as coat color and horn size [8]. Although, their productivity is much lower than that typically achieved by commercial breeds under the intensive production systems [11], indigenous cattle are often the only option available for millions of farmers in the African agro-pastoral systems, where exotic improved breeds under-perform in the traditional management systems [13].

With an extended geographic distribution across agro-ecological zones and production systems, African cattle populations represent a unique genetic resource for the understanding of the role of natural and artificial selection in the shaping of the functional diversity of a ruminant species. Moreover, unraveling their genome diversity may provide new insights into the genetic mechanisms underlying their adaptation to various agro-ecosystems [14].

In this publication, we report for the first time the genome characterization of five indigenous African cattle breeds which are representatives of the cattle diversity of the continent: N’Dama, which belong a group of West African taurine with tolerances to multiple infectious diseases; Ankole, which represents African sanga the intermediate crossbreed between zebu and taurine cattle populations, with large and distinctive horns and coat color selected by human; Boran and Kenana, two East African zebu, with beef and dairy characteristics, respectively; Ogaden, an East African zebu living in a hot and dry environment.

The comparative genome-wide analysis with three European and one Asian commercial cattle breed across African cattle types allows us to identify the unique genome response of African cattle breed to tropical challenges.

## Results and discussion

### Sequencing, assembly, and identification of single nucleotide polymorphisms

Individual genomes of 48 indigenous African (Boran, Ogaden, Kenana, Ankole, and N’Dama) cattle were generated to ~11 X coverage each and were jointly genotyped with publicly available genomes of commercial cattle breeds (Angus, Jersey, Holstein, and Hanwoo) (Fig. 1a, Additional file 1: Note S1, Table S1). These breeds comprise *Bos indicus* (Boran, Ogaden, and Kenana), African *Bos taurus* (N’Dama), European-Asian *Bos taurus*, and sanga (Ankole, cross between taurine and zebu) [8]. In total, 6.50 billion reads or ~644 Gbp of sequences were generated. Using Bowtie 2 [15], reads were aligned to the taurine reference genome sequence UMD 3.1 with an average alignment rate of 98.84% that covered 98.56% of the reference genome (Additional file 1: Table S2). Concordant with previous analysis of zebu Nellore [16], overall alignment rate of the African *B. indicus* samples to the reference genome UMD 3.1 was found comparable to the one obtained for the African taurine samples (Additional file 1: Table S2). After filtering the potential PCR duplicates and correcting for misalignments due to the presence of INDELs, we detected single nucleotide polymorphisms (SNPs) using GATK 3.1 [17]. Several filtering steps to minimize the number of false-positive calls were applied before using candidate SNPs in further analyses. In particular, SNPs were removed based on the following criteria: phred-scaled quality score, mapping quality, quality depth and phred scaled *P* value (see “Methods”). A total of ~37 million SNPs were finally retained and breed-specific SNPs were identified using SnpSift [18] (Fig. 1b, Additional file 1: Table S3). The genomic DNA from 45 African samples were additionally genotyped using the BovineSNP50 Genotyping BeadChip (Illumina, Inc.) to evaluate the accuracy of the SNP calling from the resequencing data. We observed ~95% overall genotype concordance, between the BovineSNP50 Genotyping BeadChip SNPs and the re-sequencing results across the samples, providing confidence on the accuracy of SNP calling (Additional file 1: Table S4).

### African genome diversity and relationships

#### Single nucleotide polymorphisms

Figure 1b illustrates the number of SNPs present in each breed, including breed-specific ones, with numbers provided at Additional file 1: Table S5. Looking at different cattle lineages, the largest number of SNPs is found in the zebu cattle (Boran, Kenana, Ogaden), where the great majority of the SNPs are homozygous across the three breeds representing candidate African zebu lineage specific variants. Most (65.13%) of the SNPs were present in intergenic regions. The remaining SNPs were located upstream (3.90%) and downstream (3.96%) of open reading frame, in introns (26.0%), and untranslated regions (UTRs, 0.240%). Exons contained 0.69% of the total SNPs with 115,439 missense and 1336 nonsense mutations (Additional file 1: Table S5).

**Fig. 2:**
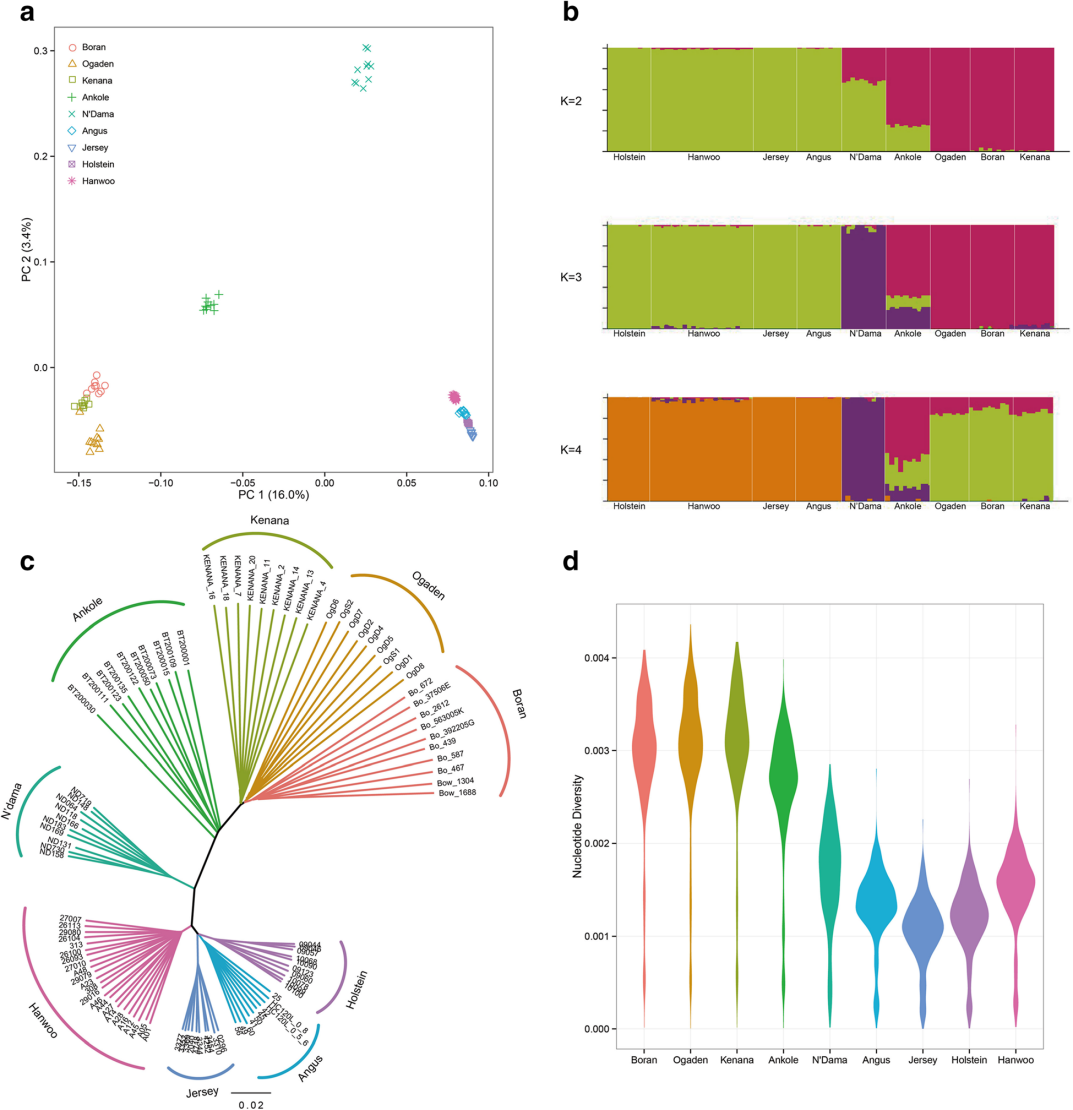
Population structure and relationships of African in comparison to commercial cattle. **a** Principal component (PC) analysis, PC 1 against PC 2. **b** Proportion of ancestry for each individual assuming different number of ancestral population (K = 2, 3, and 4). *Colors* in each *vertical line* represent the likelihood proportion of an animal genome assigned to a source population. **c** *Neighbor-joining tree* of the relationships between the nine cattle breeds (101 animals). The *scale bar* represents the identity-by-state (IBS) score between pairs of animals. **d** Genome-wide distribution of nucleotide diversity in 50-kb non-overlapping window

Nucleotide diversity measures the degree of polymorphism within a population and it is defined as the average number of nucleotide differences per site between any two DNA sequences chosen randomly from the sample population [19]. On a genome-wide window scale of 10 Mb, the commercial European breeds show reduced levels of nucleotide diversity compared to all indigenous African breeds (Fig. 2d). Here, the reduced level of nucleotide diversity at the whole-genome level is expected and is likely the result of intensive artificial selection over generations and/or genetic drift followed by a demographic history characterized by a low effective population size. Interestingly, N’Dama also show relatively low genetic diversity, perhaps a legacy of an initial low effective population size and/or of population bottleneck following disease challenges [20]. Nucleotide diversity is the highest across the African zebu (Boran, Ogaden, Kenana) and the Ankole sanga. These are admixed taurine × zebu breeds with a relatively large effective population size. The relatively high nucleotide diversity in the commercial Hanwoo may reflect weaker, targeted, and shorter selection history compared to other commercial breeds [21].

#### Population structure and relationships

We performed principal component analysis (PCA) of the autosomal SNPs genotype data (Fig. 2a) using EIGENSTRAT [22]. The analysis ignores breed membership but nevertheless reveals clear breed structures as samples from the same breed cluster together. The first two PCs, explaining 16.0% and 3.4% of the total variation, respectively, separate African from non-African breeds with the Ankole cattle at an intermediate position. PCA based on African, commercial, and taurine samples separately (Additional file 1: Figure S1) show no evidence of admixture between breeds or the presence of outlier animals within breeds.

To further understand the degree of admixture in the populations, we used STRUCTURE [23, 24] on a randomly sampled subset of SNPs (~20,000 SNPs). We increased *K* from 1 to 9, where *K* is the assumed number of ancestral populations (Fig. 2b and Additional file 1: Figure S2). The analysis suggested *K* = 2 as the most likely number of genetically distinct groups within our samples (Fig. 2b), reflecting the divergence of taurine and zebu cattle in the cattle population. At K = 3, Ankole showed clear evidence of genetic heterogeneity with shared genome ancestry with African (N’Dama), Asian zebu, and commercial (Holstein, Jersey, Angus, Hanwoo) taurine genetic background. Increasing values of *K* indicated higher levels of breed homogeneity in the commercial population compared to African zebu breeds. In addition, a neighbor-joining tree (Fig. 2c) separates each breed in its own separate clade. European breeds cluster together, then with the Hanwoo and the N’Dama. Similarly, all the African zebu breeds cluster together and Ankole animals are found at an intermediate position between zebu and N’Dama.

#### Demographic history and migration events

**Fig. 3:**
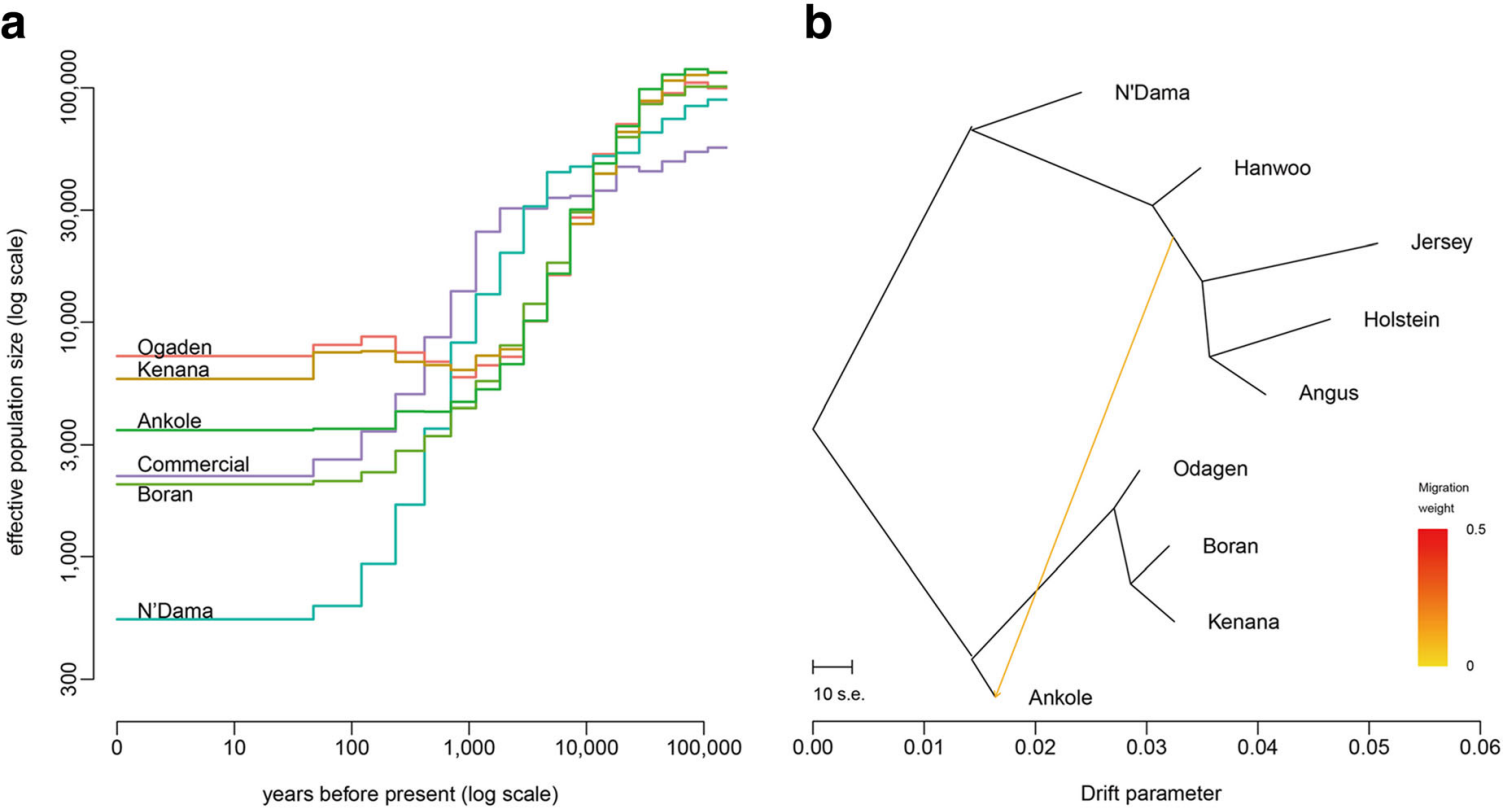
African cattle effective population size and history. **a** Estimated effective population size of each African cattle breed and the combined commercial (Hanwoo + Jersey + Holstein + Angus). **b** Pattern of population splits and mixture between the nine cattle breeds. The drift parameter is proportional to *Ne* generations, where *Ne* is the effective population size. *Scale bar* shows ten times the average standard error of the estimated entries in the sample covariance matrix. The migration edge from the European taurine lineage into the Ankole is colored according to the percent ancestry received from the donor population

Variation of effective population size through time [25] is shown in Fig. 3a and Additional file 1: Figure S3. N’Dama seemed to have suffered a stronger population decline compared to the other African populations. This observation is compatible with an initial population bottleneck following the arrival and adaptation of the ancestral population in the tropical sub-humid and humid Western African environment. These West African cattle populations have been subjected in recent times to new environmental pressures imposing strong adaptive constraints (e.g. new pathogens including parasites) [26, 27]. In addition, the estimates of Ogaden and Kenana show a slight increase in population size around 1000 years ago corresponding to the time of the first wave of zebu arrival through the Horn of the continent [8]. All share a common population decline starting approximately 10,000 BP, a likely consequence of Neolithic domestication events [25, 28].

We then reconstructed the maximum likelihood tree (Fig. 3b) and residual matrix (Additional file 1: Figure S4) of the nine breeds using Treemix [29] to address population history relationships and to identify pairs of populations that are related to each other independent of that captured by this tree. Adding sequentially migration events to the tree, we found that one inferred migration edge produces a tree with the smallest residuals and thus best fits the data (Additional file 1: Figure S4). We observed a statistically significant migration edge (*P* < 2.2E-308) with the estimated weight of 11.4%; this edge provides evidence for the gene flow from European *B. taurus* (represented here by Jersey, Holstein, and Angus) into Ankole. In recent years, Ankole cattle have been increasingly crossbred with taurine breed including Holstein cattle which were first introduced to Uganda 50 years ago [30].

### The adaptation of African cattle to environmental stresses and human selection

We compared the genomes of African cattle breeds to identify within each breed signatures of positive selection following environmental and human selection pressures. In contrast to SNP chip data, where diversity is overestimated in taurine lineages and underestimates in indicine lineages [31], whole-genome sequencing can overcome this limit of ascertainment bias to properly enable population analyses of both populations and to identify targets of selections in African *B. indicus* as well. In particular, we examined extreme haplotype homozygosity and allele frequency differentiation over extended linked regions using cross-population extended haplotype homozygosity (XP-EHH) [32] and the cross-population composite likelihood ratio (XP-CLR) [33]. Considering the close genetic distance among African *B. indicus* (Additional file 1: Table S6), N’Dama and Ankole cattle breeds were separately compared against all other African breeds for the identification of African breed-specific signatures. XP-EHH maintains power with small sample size (as low as ten samples) [34]. In addition, when estimates of genetic distance (*F*_*ST*_) between pairs of populations are greater than or close to 0.05, as in our analyses (Additional file 1: Table S6), fewer than 20 individuals per population should be sufficient for population differentiation analysis [35]. To enable comparisons of genomic regions across populations, we divided the genome into non-overlapping segments of 50 Kb [36]. Outlier regions (the top 0.5% XP-EHH or XP-CLR statistics) were considered to be breed-specific candidate regions for further analysis (haplotypes and polymorphisms). The distributions of raw XP-EHH and XP-CLR values of each comparison and SNP density in each non-overlapping 50-kb window are provided in Additional file 1: Figures S5–S7.

#### The adaptation of N'Dama to trypanosome challenge

We first investigated how tolerance to trypanosome challenge may have impacted the genome of African cattle. African trypanosomes are extracellular protozoan parasite that cause severe diseases in human (sleeping sickness) and domestic animals (nagana); approximately 60 million people and 50 million cattle are living at risk of trypanosome infection [37, 38]. Among a few “trypanotolerant” indigenous African cattle breeds, the West African N’Dama is the best characterized, while the “newcomer” *B. indicus* are generally highly susceptible to trypanosomosis [39]. We therefore compared the N’Dama genome against all other African cattle breeds.

Outlier windows from XP-EHH and XP-CLR analysis include 124 and 106 genes, respectively, 28 of which were common to both analyses (Table 1, Additional files 2 and 3). This relatively modest overlap likely resulted from difference in power between the tests designed to detect regions affected by complete (XP-EHH) or incomplete selective sweeps (XP-CLR).

**Table 1:** Summary of major candidate regions identified from XP-EHH and XP-CLR in each breed comparison (see Additional files 2 and 3 for summary values of all candidate genes)

Gene	CHR^a^	Max XP-EHH^b^	XP-EHH *P* value^c^	XP-CLR	Association	Candidate SNP position	Selected breed
*HCRTR1*	2	-	-	597.3	Circadian rhythm, feeding behavior		N’Dama
*STOM*	8	-	-	525.0	Anemia	112665146 (p.Met48Val)	N’Dama
*SLC40A1*	2	3.32	0.0002	831.1	Anemia	-	N’Dama
*SBDS*	25	2.91	0.0024	-	Anemia	-	N’Dama
*EPB42*	10	-	-	511.1	Anemia	38523031 (p.Arg503His)	N’Dama
*RPS26*	6	-	-	562.8	Anemia	-	N’Dama
*KIT*	6	1.80	0.0050	-	Coat color	-	Ankole
*MITF*	22	1.90	0.0032	-	Coat color	-	Ankole
*PDGFRA*	6	2.56	0.0001	319.3	Coat color	-	Ankole
*FGF18*	20	-	-	182.3	Horn development	-	Ankole
*MC1R*	18	-	-	295.0	Coat color		Ankole
*SOD1*	1	-	-	333.31	Thermoregulation	3116044 (p.Ile95Phe)	*B. indicus*
		-	-	186.33			African
*PRLH*	3	1.49	0.0014	-	Thermoregulation	117646610 (p.Arg76His)	*B. indicus*
		1.17	0.0039	-			African
*BOLA*	23	1.19	0.003	110.13	Tick resistance	-	African

Dash (–) indicates non-significant results

^a^Chromosome

^b^Maximum (positive) XP-EHH score of all SNPs within a window

^c^Rank-based empirical *P* value of genomic region

**Fig. 4:**
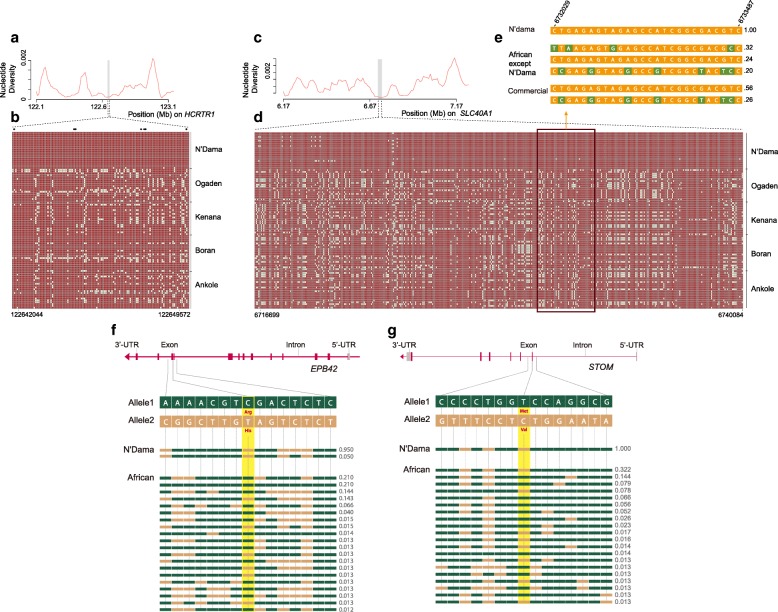
Signatures of selective sweep at the N’Dama *HCRTR1*, *SLC40A1*, *EPB42*, and *STOM* gene regions. Nucleotide diversity plots of the *HCRTR1* (**a**) and *SLC40A1* (**c**) genomic regions. Haplotype diversity at the *HCRTR1* (**b**) and *SLC40A1* (**d**) gene regions (*gray area*). The major allele at each SNP position in N’Dama is colored in *red*, the minor one in *white*. The *star* (*) denotes non-synonymous N’Dama SNP identified at the *HCRTR1* gene region. **e** Frequency of N’Dama fixed haplotype (SLC40A1 region) in others breeds with comparison with major observed haplotype(s) (frequency > 0.15 shown). Nucleotide with *green* background represents distinct polymorphism compared to the major SNP allele present in N’Dama. **f**, **g** Structure of the *EPB42* and *STOM* gene with exons indicated by *vertical bars*. Non-synonymous SNPs represent p.Arg503His and p.Met48Val and are highlighted in *yellow*. Different color represents different alleles, and frequency of each haplotype is indicated on the *right side* of the figure

Among these, we found *HCRTR1* (XP-CLR = 597.3) encoding hypocretin receptor A (Fig. 4), which belongs to the class I subfamily within the superfamily of G-coupled receptors and is coupled to Ca^2+^ mobilization. Hypocretins are produced by a small group of neurons in the lateral hypothalamic and perifornical areas and they are involved in the control of mammalian feeding behavior [40]. Compared to other African cattle, N’Dama show almost pure haplotype homozygosity at the *HCRTR1* region and we also detect seven non-synonymous variants in the gene (Fig. 4b) (Additional file 1: Table S7). Numerous studies indicate that polymorphism within hypocretin genes are associated with alterations in feeding and drinking behaviors [41, 42]. In particular, orexin-A, endogenous ligands for G protein coupled receptor, stimulated food consumption, and orexin messenger RNA is upregulated by fasting [43]. These independent studies indicate that the hypocretins have a major role in the regulation of feeding. It may explain the superior ability of N’Dama to maintain body weight and resist listlessness and emaciation following trypanosome infection [44, 45].

N’Dama cattle achieve trypanotolerance with at least two additional characteristics: the ability to resist anemia and to control parasite proliferation [46–48]. Anemia is the most prominent and consistent clinical sign of *Trypanosoma* infection and it is the main indicator for treatment [49]. We found five genes within genome regions putatively positively selected (outlier windows) that are associated with anemia (*SLC40A1*, *STOM, SBDS*, *EPB42*, and *RPS26*). The iron exporter SLC40A1 (XP-EHH = 3.32, XP-CLR = 831.1) is essential for iron homeostasis and it is therefore related to iron-deficiency anemia [50]. This gene shows a local reduction in nucleotide diversity and extended haplotype pattern (Fig. 4c). Notably, we found a fixed SLC40A1 haplotype in N’Dama, with frequency of 24% and 58% in other African cattle and commercial breeds, respectively, strongly supporting selection at the gene (Fig. 4d, e). Stomatin (*STOM*, XP-CLR = 525.0) is a gene named after a rare human hemolytic anemia [51], and it encodes a 31-kDa integral membrane protein. Mutations in *SBDS* (XP-EHH = 2.91) *EPB42* (XP-CLR = 511.1) genes are responsible for hypochromic anemia [52] and hereditary hemolytic anemia [53], respectively, while mutations at *RPS26* (XP-CLR = 562.8) gene have been identified in Diamond-Blackfan anemia patients [54–56].

We further screened these candidate genes for non-synonymous mutations representing putative functional variants. Notably, missense SNPs changed amino acids in the STOM (p.Met48Val) and EPB42 (p.Arg503His) proteins. Both of these allele variants are completely fixed in N’Dama cattle in contrast to all the other breeds (Fig. 4f and g).

Genes positively selected in N’Dama were significantly (*P* < 0.05) over-represented in “I-kappaB kinase/NF-kappaB cascade” (GO:0007249, Additional file 4). The transcription factor nuclear factor-kappaB (NF-kB) is central to the innate and acquired immune response to microbial pathogens, coordinating cellular responses to the presence of infection. In fact, based on the molecular evidence that *Trypanosoma cruzi* activates NF-kB in a number of cells, NF-kB was suggested as a determinant of the intracellular survival and tissue tropism of *T. cruzi*, which causes human sleeping sickness [57]. These studies may suggest that genes involved in NF-kB cascade have experienced positive selection in N’Dama to alter in functions to effectively regulate the infection of cattle trypanosome. We also found a significant signal at interleukin 1 receptor-like 2 (*IL1RL2*) in agreement with the observation that the initial response of the host immune system to trypanosomes infection includes activation of macrophages secreting pro-inflammatory molecules such as IL-1 [58, 59]. In particular, it has been previously reported that *T. brucei* infections result in increase of IL-1 secretion [60].

#### The impact of human selection on Ankole genome

In the Ankole versus all other African cattle comparisons, we identified 187 genes within the outlier genome windows (Table 1, Additional files 2 and 3). The putatively selected genomic regions include candidate loci that have biological functions related to coat color: melanocortin 1 receptor (*MC1R*) (XP-CLR = 295.0) and *KIT* (XP-EHH = 1.80), both of which are supported by haplotype sharing analysis showing high level of haplotype homozygosity within the breed (Additional file 1: Figure S8). Ankole cattle are characterized by their massive white horns and predominantly red coat color [61]. The results are in agreement with previous reports that mutations in *MC1R* generate red (or chestnut) coat colors in various species including cattle, horses, mice, and dogs [62, 63]. The product of *KIT* is likely involved in the white spotting of the coat, not only in cattle but also in other domesticated mammals [64]. Our findings are consistent with the observation that while the coat color of Ankole is predominantly red, it is also sometimes white-spotted [61]. Interestingly, Holstein, also known for their black-and-white markings, share the same haplotype (Additional file 1: Figure S8) in the *KIT* gene region as the one observed in Ankole, indicating a common origin of the haplotype in the African and European taurine lineages and/or recent crossbreeding of Ankole with Holstein cattle. We also found *MITF* (XP-EHH = 1.90) and *PDGFRA* (XP-EHH = 2.56, XP-CLR = 319.3) genes within the outlier regions; these were previously also associated with white spotting in various dairy cattle breeds and other species [65–67] (Table 1, Additional files 2 and 3).

We also found putative candidate selected regions that might have shaped the massive horn in Ankole. We initially evaluated a previously reported candidate variant responsible for the presence of horns in Holstein [68]. All Ankole samples showed the genotype G/G at BTA1:1390292G > A indicating that Ankole followed the genotype of horned Holstein cattle [68]. Over-representation analysis of gene ontology (GO) terms (Additional file 4) shows that Ankole has increased GO categories involved in fibroblast growth factor (FGF) signaling pathway (*MAP3K5*, *PPP2R2C*, *FGF18*, and *FRS3*, P00021) and skeletal system development *ACVRL1*, *CASR*, *TLX3*, *ACVR1B*, and *RUNX3*, GO:0001501). Neither term was enriched from positively selected genes in any other African cattle, indicating they may therefore be linked to the extreme horn development observed in the breed. Horn is an outgrowth of the frontal bone covered by a tough shell of modified epithelium, derived from dermal and subcutaneous connective tissue [69, 70]. FGF signaling pathway includes *FGF18* (XP-CLR = 182.3), which is responsible for differentiating osteoblasts during calvarial bone development [71] and is associated with chondrocyte proliferation [72] in mouse. These genes together might underlie the distinctive morphology of Ankole horn versus other cattle.

#### The adaptation of African cattle to tick challenges

African cattle breeds have evolved to adapt to the harsh environmental conditions prevailing across sub-Saharan Africa such as tropical livestock diseases, high solar radiation and temperature, drought, and poor nutritional condition [73, 74]. These environmental conditions prevail across sub-Saharan Africa and a signal of positive selection may be expected to be common across African breeds. To investigate this, all African breeds were combined and compared to the commercial breeds for the identification of common and unique African genome specific signature of selection. In this comparison, XP-CLR and XP-EHH analyses reveal outlying windows (top 0.5%) with 252 genes (Additional files 2 and 3). Among these, we found the region including the bovine lymphocyte antigen (*BOLA*, XP-EHH = 1.19, XP-CLR = 110.1) gene. Examining the region in details we identified six *BOLA* haplotype blocks where major African cattle haplotypes correspond to contrasting or the minor haplotypes in commercial cattle (Additional file 1: Figure S9). Alleles of BOLA-DRB3 showed association with resistance to tick (*Boophilus microplus*) infestation in cattle [75]. The bovine lymphocyte antigen complex has been studied extensively for the past 30 years because of its importance in host immunity [76]. Most studies have focused on other BOLA family members and their relevance to parasitic diseases and thus elucidating the function of this *BOLA* gene in African cattle may unravel the mechanisms behind the interaction between *BOLA* complex and the innate immunity against several important tropical parasitic diseases such as East Coast Fever [77].

#### Heat tolerance in African cattle

**Fig. 5:**
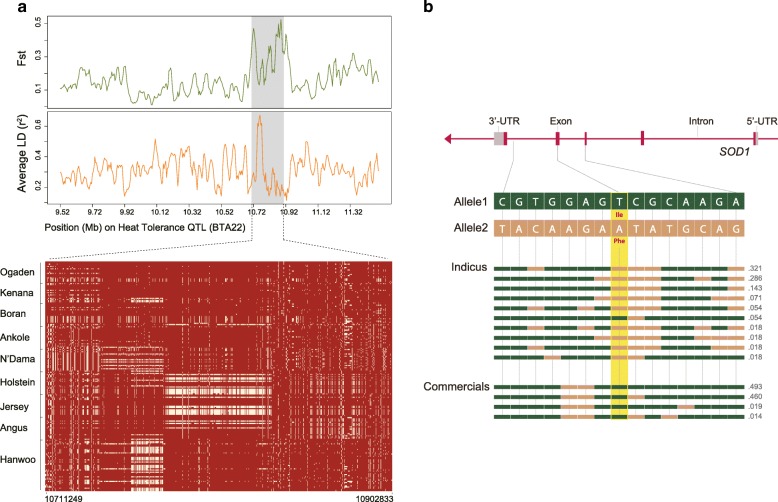
A selective sweep associated with heat tolerance in African cattle. **a** Fixation index (*Fst*) and linkage disequilibrium values for *Bos indicus* samples in 20-kb sliding windows with 5-kb steps (*top*) and the degree of haplotype sharing around heat tolerance QTL (10.71–10.90 Mb region on chromosome 22). *Fst* is calculated between *B. indicus* and commercial samples*.* The major allele in each *B. taurus* and *B. indicus* populations is indicated in *red*. **b** Structure of the *SOD1* gene with exons indicated by *vertical bars*. A non-synonymous SNP represents p.Ile95Phe and is highlighted in *yellow*. Haplotype frequencies are indicated by numbers next to each haplotype. In each haplotype, *green* and *beige bars* represent alleles 1 and 2, respectively

To identify genomic regions responsible for thermoregulation in African cattle, we selected a priori candidate genes by using 13 previously identified heat tolerance quantitative trait loci (QTL) regions [78] and 18 heat shock proteins. None of these regions were supported by our common metrics of XP-EHH and XP-CLR. We then analyzed the pattern of haplotype homozygosity in African cattle compared to the European and Asian taurine (commercial breeds developed in temperate areas). Consistent with our previous results, we found haplotype sharing to be much more extensive in the commercial breeds when random genomic regions were examined (Additional file 1: Figure S10). However, looking at the candidate regions in African breeds in comparison to the commercial ones, remarkable long-range haplotypes are shared across African cattle within one of the heat tolerance QTLs (BTA22, 10.03–11.0 Mb) (Fig. 5a) and in one of heat shock proteins, heat shock 70 kDa protein 4 (*HSPA4*) (Additional file 1: Figure S11), indicative of selective sweeps for heat tolerance in this region. Cellular tolerance to heat stress is mediated by a family of heat shock proteins. Heat shock protein 70 is notable for promoting cell protection against heat damage and preventing protein denaturation [79, 80]. The degree of haplotype sharing at these two regions was noted to be more extensive in *B. indicus* African cattle than in the N’Dama*,* which is consistent with a previous report that zebu breeds are better able to regulate body temperature in response to heat stress [81]. The heat tolerance QTL region identified here is further supported by multiple signatures of positive selection within *B. indicus* populations showing elevated linkage-disequilibrium and high population divergence (*Fst*) compared to the taurine breeds (Fig. 5a).

We also found a strong signal for positive selection at the superoxide dismutase 1 (*SOD1*, XP-CLR = 333.3) gene (Additional file 3) in both African versus commercial breeds and *B. indicus* versus commercial breeds comparisons. Okado-Matsumoto and Fridovich [82] have shown that binding of heat shock proteins to mutant forms of protein abundant in motor neurons, such as SOD1, makes heat shock proteins unavailable for their antiapoptotic functions. Considering that *B. indicus* cattle are better adapted to higher ambient temperature, and the selection signal was stronger in *B. indicus*, further comparisons were made between *B. indicus* and commercial breeds only. Functional annotation of variants located in this gene identified a missense mutation (p.Ile95Phe) in exon 3 of SOD1 only in *B. indicus* population. This non-synonymous mutation, in contrast to the pattern observed in commercial breeds, has nearly reached fixation (95%) in the zebu populations (Fig. 5b). These results suggest that variations in *SOD1* gene may play an important role in heat tolerance traits observed African cattle.

A recent study has expanded the scope of classical prolactin biology [83]. It shows that the prolactin signaling pathway is involved not only in lactation but also has an impact on hair morphology and thermoregulation phenotypes in the predominantly taurine Senepol cattle. This is most likely mediated by two reciprocal mutations in the prolactin (*PRL*) and its receptor (*PRLR*) genes [83]. Analyzing all African cattle together in comparison to commercial breeds, a significant selection signal, stronger if only *B. indicus* are examined (Table 1), was found in the prolactin releasing hormone (*PRLH*, XP-EHH = 1.49) gene region which stimulates prolactin release and regulates the expression of prolactin. We then observed that one non-synonymous SNP in exon 2, which encodes a p.Arg76His substitution, is highly conserved in the *B. indicus* cattle population (73%) and absent in the commercial taurine (Additional file 1: Figure S12). These results together suggest that the *PRLH* mutation may confer a selective advantage in regulating prolactin expression which might be linked to the thermotolerance in African cattle, especially in *B. indicus*.

Our GO analysis (Additional file 4) revealed the most significant enrichment of Wnt signaling (P00057) as well as pathways involved in regulating skin blood flow: endothelin signaling pathway (P00019) and histamine H1 receptor mediated signaling pathway (P04385). Thermoregulatory control of skin blood flow is vital to the maintenance of normal body temperatures during challenges to thermal homeostasis [84] and, specifically, the rise in skin blood flow during body heating contains an H1 histamine receptor component [85]. These pathways might be rapidly evolving in African cattle, which might explain their completely different degree of thermotolerance at the cellular and physiological levels compared to temperate cattle breeds.

## Conclusion

In this study, we have generated for the first time a catalog of genetic variants found in selected sub-Saharan African cattle. While the studied breeds represent only a small subset of the 150 recognized on the African continent [86], they illustrate the extraordinary diversity present within and across African cattle breeds. We were able to highlight and map at the genome level some unique African adaptations, which may represent responses to climatic challenges (e.g. heat), disease resistance (e.g. trypanosomosis challenge), and artificial selection (e.g. coat color, horn development), including new genes or gene pathways putatively involved in these adaptations. These results can therefore inform targeted genomic in vitro, and in vivo studies to further test hypotheses arising from our work, and to identify underlying genomic mechanisms. On a practical note, these results provide new genomic evidence and options for designing and implementing genetic intervention strategies for improved cattle productivity and resilience in sub-Saharan Africa. Already, some of our results may provide new avenues for the improvements of livestock productivity and resilience to environmental challenges within breeds and through crossbreeding (e.g. marker-assisted selection to increase haplotypes frequencies). Perhaps most importantly, this study shows the value of comparative genome studies in cattle breeds selected for diverse environments and it argues for the value of a comprehensive continent-wide characterization of the genome landscape of African cattle. The African continent is now witnessing major transformations of its agricultural systems and rapid loss of indigenous livestock. Unfortunately, the opportunity to explore this treasure trove of diversity may not last for very much longer.

## Methods

### Samples and DNA re-sequencing data

Whole-blood samples (10 ml) were collected from ten Ankole, ten Boran, nine Kenana, ten N’Dama, and nine Ogaden cattle. We generated pair-end reads using Illumina HiSeq2000. DNA was isolated from whole blood using a G-DEXTMIIb Genomic DNA Extraction Kit (iNtRoN Biotechnology, Seoul, Republic of Korea) according to the manufacturer’s protocol. We randomly sheared 3 μg of genomic DNA using the Covaris System to generate inserts of ~300 bp. The fragments of sheared DNA were end-repaired, A-tailed, adaptor ligated, and amplified using a TruSeq DNA Sample Prep. Kit (Illumina, San Diego, CA, USA). Paired-end sequencing was conducted using the Illumina HiSeq2000 platform with TruSeq SBS Kit v3-HS (Illumina). Finally, sequence data were generated using the Illumina HiSeq system.

Incorporating our previously published data of 53 commercial breed samples (Additional file 1: Table S1), we performed a per-base sequence quality check using the fastQC software (http://www.bioinformatics.bbsrc.ac.uk/projects/fastqc/). The pair-end sequence reads were then mapped against the reference bovine genome (UMD 3.1) using Bowtie2 [15]. We used default parameters (except the “--no-mixed” option) to suppress unpaired alignments for paired reads. The overall alignment rate of reads to the reference sequence was 98.84% with an average read depth of 10.8×. On average across the whole samples, the reads covered 98.56% of the reference UMD3.1 genome (Additional file 1: Table S2).

We used open-source software packages for downstream processing and variant calling. Using the “REMOVE_DUPLICATES = true” option in “MarkDuplicates” command-line tool of Picard (http://broadinstitute.github.io/picard), potential PCR duplicates were filtered. We then used SAMtools [87] to create index files for reference and bam files. Genome analysis toolkit 3.1 (GATK) [88] was used to perform local realignment of reads to correct misalignments due to the presence of indels (“RealignerTargetCreator” and “IndelRealigner” arguments). The “UnifiedGenotyper” and “SelectVariants” arguments of GATK were used for calling candidate SNPs. To filter variants and avoid possible false positives, argument “VariantFiltration” of the same software was adopted with the following options: (1) SNPs with a phred-scaled quality score < 30 were filtered; (2) SNPs with MQ0 (mapping quality zero; total count across all samples of mapping quality zero reads) > 4 and quality depth (unfiltered depth of non-reference samples; low scores are indicative of false positives and artifacts) < 5 were filtered; and (3) SNPs with FS (phred-scaled *P* value using Fisher’s exact test) > 200 were filtered since FS represents variation on either the forward or the reverse strand, which are indicative of false-positive calls.

We additionally genotyped 45 cattle samples (of which blood samples were available) using BovineSNP50 Genotyping BeadChip (Illumina, Inc.). After filtering out SNPs based on GeneCall score < 0.7, common loci of SNP chip and DNA resequencing data were extracted and examined to assess concordance (Additional file 1: Table S4).

We used BEAGLE [89] to infer the haplotype phase and impute missing alleles for the entire set of cattle populations simultaneously. A summary of the total number of SNPs identified is provided in Additional file 1: Table S3. Sequences are available from GenBank with the Bioproject accession number PRJNA312138.

### Identification of breed-specific enriched SNPs using SnpSift

We performed enrichment analysis to identify breed-specific SNPs using SnpSift [18]. In SnpSift, several statistical tests are implemented such as Fisher’s exact test and Cochran–Armitage trends test for analyzing genotype count data composed with two factors. Generally, one of the factors is fixed as the genetic model, which can be dominant, recessive, or co-dominant. The other is breed information, which was employed in this study for identifying breed-specific enriched SNPs. We had nine different cattle breeds that were used for breed-specific versus the others. From these two factors, we have constructed 2 × 2 (dominant or recessive coding/breed-specific group information) or 2 × 3 (co-dominant coding/breed-specific group information) contingency tables. As a result, three different contingency tables are generated for each breed and SNPs. We performed Fisher’s exact test and Cochran–Armitage trend test for the 2 × 2 and 2 × 3 contingency tables, respectively. A total of 37,460,739 SNPs were employed in the test; this induces multiple testing problems. To correct the multiple testing errors, we used Bonferroni correction, which is the most conservative method. After discovering significant breed-specific enriched SNPs, we annotated each SNP using snpEff. We focused on the non-synonymous SNPs (MISSENSE and NONSENSE) for this annotation.

### Statistics to explore selective sweep regions in African cattle

To uncover genetic variants involved in local adaptation of each breed group, we performed comparisons between populations: (1) N’Dama versus all other African, (2) Ankole versus all other African; (3) all African versus all commercial; and (4) all *B. indicus* versus all commercial. The XP-EHH method was first used to detect selective sweeps using the software xpehh [32] (http://hgdp.uchicago.edu/Software/). This statistic detects haplotypes in one of the populations that have increased in frequency to the point of complete fixation [32, 34]. An XP-EHH raw score distribution plot is provided in Additional file 1: Figure S5. We then split the genome into non-overlapping segments of 50 kb to use the maximum (positive) XP-EHH score of all SNPs within a window as a summary statistic for each window. To consider the variation in SNP density, we binned genomic windows according to their numbers of SNPs in increments of 500 SNPs (combining all windows ≥ 1000 SNPs into one bin). A histogram of SNP density in each window is provided in Additional file 1: Figure S6. Within each bin, for each window *i*, the fraction of windows with a value of the statistic greater than that in *i* is defined as the empirical *P* value, following the method previously reported [34, 90]. The regions with *P* values less than 0.005 (0.5%) were considered significant signals in the breed group of interest. This approach is suitable, especially with the unreliable demographic model and parameters, as is the case for cattle [34]. However, loci detected as being under selection using this approach may under-represent selection on standing variation [91].

We additionally performed the XP-CLR [33] to search for regions in the genome where the change in allele frequency at the locus occurred too quickly, which is assessed by the size of the affected region. XP-CLR scores were calculated using scripts available at (http://genetics.med.harvard.edu/reich/Reich_Lab/Software.html). We used the following options: non-overlapping sliding windows of 50 kb, maximum number of SNPs within each window as 600, and correlation level of 0.95 from which the SNPs contribution to the XP-CLR result was down-weighted. The regions with the XP-CLR values in the top 0.5% of the empirical distribution were designated as putative selective sweeps.

“Significant” genomic regions identified from XP-EHH and XP-CLR tests were annotated to the closest genes (UMD 3.1). Genes that overlap the significant window regions were defined as candidate genes. PANTHER (version 11.0) [92] was used to determine if there was any significant over-representation of genes with particular functional categories (GO-slim Biological Process and PANTHER pathways), that is, functional enrichment, among positive selected genes in each African breed. *P* value of 0.05 (no correction for multiple testing) was used as the criterion for statistical significance. Heat tolerance trait QTL from the animal QTL database (Animal QTLdb, http://www.animalgenome.org/cgi-bin/QTLdb/index) were defined by a trait class of “Heat tolerance” [93].

### Population differentiation and structure

For PCA, we used genome-wide complex trait analysis (GCTA) [94] to estimate the eigenvectors, which is asymptotically equivalent to those from the PCA implemented in EIGENSTRAT [22], incorporating genotype data from all samples. For admixture analysis, we restricted the genotype data to a random subset of ~ 20,000 of total SNPs using PLINK (--thin option) [95] to run the “admixture” model in STRUCTURE version 2.3 [23]. We chose 20,000 iterations after a burn-in of 50,000 iterations and the analysis was repeated ten times for each K value. The output of STRUCTURE was then analyzed in STRUCTURE HARVESTER [96], which implements the Evanno method to infer the most likely number of clustered populations. We used VCFtools 4.0 [97] to estimate nucleotide diversity (in windows of 10 Mb) and the *Fst* divergence statistic with the VCFtools implementation of *Fst* and weighted *Fst* estimators as described in Weir and Cockerham [98] for each pair of populations. Linkage disequilibrium between pairs of markers were assessed using PLINK [95]. The *r* ^*2*^ value was calculated between all pairs of SNPs with inter-SNP distances of less than 20 kb (*r* ^*2*^ and ld-window parameters). Moving averages (sliding windows) of the pairwise LD coefficients were then carried out in 20-kb windows with 5-kb steps). We used Haploview software [95] to evaluate the haplotype structure and estimate haplotype frequencies.

### Phylogenetic reconstruction and inference of demographic history

A neighbor-joining tree was constructed with FigTree v1.4.0 on the basis of the IBS distance matrix data of all samples calculated by PLINK [95]. We also inferred a population-level phylogeny using the maximum likelihood (ML) approach implemented in TreeMix [29]. The window size of 1000 was used to account for linkage disequilibrium (-k) and “-global” to generate the ML tree. Migration events (-m) were sequentially added to the tree. The recent demographic history was inferred by the trend in effective population size (Ne) change using PopSizeABC [25] with default parameters set for cattle population (mutation rate and recombination of 1e-8, MAF > 20%, size of each segment = 2,000,000) and simulated 50,000 datasets. The 90% credible intervals associated with the estimated population size histories for each breed are shown in Additional file 1: Figure S3.

## Additional files

Additional file 1: Supplemental data including Note S1, Tables S1-S7 and Figures S1-S12. (DOCX 14235 kb)
Additional file 2: Summary of XP-EHH analysis. (XLSX 43 kb)
Additional file 3: Summary of XP-CLR analysis. (XLSX 37 kb)
Additional file 4: Summary of Gene Ontology enrichment analysis. (XLSX 17 kb)
