# Supplementary material for: The genome landscape of indigenous African cattle
Source: Genome Biol. 2017 Feb 20;18:34. doi: 10.1186/s13059-017-1153-y (PMC5319050; doi:10.1186/s13059-017-1153-y)

**The genomic landscape of indigenous African cattle**

Jaemin Kim*, Olivier Hanotte*, Okeyo Ally Mwai, Tadelle Dessie, Salim Bashir, Boubacar Diallo, Morris Agaba, Woori Kwak, Samsun Sung, Minseok Seo, Hyeonsoo Jeong, Taehyung Kwon, Kwondo Kim, Mengistie Terefe, Seoae Cho, Hyun-Jeong Lee, Duhak Yoon, Sung Jong Oh, Stephen Kemp, Hak-Kyo Lee*, Heebal Kim*

| **Additional file 1** |
| --- |

1. **Additional file 1: Note………………………………………………………... 2**
2. **Additional file 1: Tables**

**Table S1…………………………………………………….. 6**

**Table S2…………………………………………………….. 7**

**Table S3…………………………………………………….. 9**

**Table S4…………………………………………………….. 10**

**Table S5…………………………………………………….. 12**

**Table S6…………………………………………………….. 13**

**Table S7…………………………………………………….. 14**

1. **Additional file 1: Figures**

**Figure S1…………………………………………………… 15**

**Figure S2…………………………………………………… 16**

**Figure S3…………………………………………………… 17**

**Figure S4…………………………………………………… 18**

**Figure S5…………………………………………………… 19**

**Figure S6…………………………………………………… 20**

**Figure S7…………………………………………………… 21**

**Figure S8…………………………………………………… 22**

**Figure S9…………………………………………………… 23**

**Figure S10………………………………………………….. 24**

**Figure S11………………………………………………….. 25**

**Figure S12………………………………………………….. 26**

**Note S1.** Characteristics of the African cattle and commercial breeds.

The **Ankole** and its related strains (e.g. Watusi, Kigezi) are sanga type of cattle. They are ancient crossbreed *Bos indicus* and *B.* *taurus* cattle populations. They are found in Uganda, Rwanda, Burundi and Tanzania^1-5^. These cattle have a medium body size with small birth weight, characteristic elegant gait, very long and symmetrical horns, a relatively short neck, weak legs, and solid brown color for their preferred coat color pattern[^1^](#_ENREF_1). They are well adapted to hot climate being able to use and survive on poor quality feed and withstand severe droughts. The breed is tolerant against ticks and display resistance to theileriosis and helminthes^5^. We sequenced Ankole cattle from Uganda.

The **N’Dama** is a taurine cattle distributed in tsetse-infested regions of West and Central Africa. It is compact with short legs of fine bone and therefore small body size; neck is thick and deep. The coat color is predominantly red or black^1^. It is known for its trypanosomosis resistance^6,7^ and it also shows superior resistance to several parasitic diseases and/or infections, including helminths^1^. The animals studied were sampled in the Fouta Djallon area of Guinea, the geographic center of origin of the breed^1^.

**Boran** cattle belong to the large East African Zebu breeds of cattle. The Boran cattle likely originate in Ethiopia and today they are found in this country as well as in Kenya (Kenyan Boran, Orma Boran) and Somalia^1,4^. The Kenyan Boran is a meat type of breed. They are usually white or grey fawn, the hooves and muzzle are always black^1^. Boran cattle have the reputation to be very versatile and to adapt well to various environments^1^. They have the ability to thrive well in dry and low rainfall areas. The breed can walk long distances in hot climate in search of feed and water. The animal studied here are Kenya Boran from the ILRI Kapiti Ranch.

The **Kenana** breed is zebu cattle breed largely under the custody of semi-nomadic pastoralists in northern Sudan. They are medium to large in size. Coat color is white or steel grey with darker shading on the neck and shoulders and a black switch to the tail; blue-grey to white with black shadings on the head, neck hump, hindquarters and legs; horns are black. Calves are born ‘’red’’ in color turning white/grey when they get older. Horns are often loose; females have shorter horns than males^1,4,8^. Kenana cows are considered as one of the best African zebu milk breed[^1^](#_ENREF_1). Our animals were sampled in Sudan, and they were collected from different herds around the city of Rabak located approximately 260 kilometers south of Khartoum.

**Ogaden** cattle studied here originate from the Ogaden area in the southeastern portion of the Somali Regional State in eastern Ethiopia. Their coat color pattern is uniform plain white. However, some animals have shades of black around the face, hump and rump. They are similar to the Ethiopian Boran with well-developed hump and large dewlap; horns are short[^1^](#_ENREF_1).

**Holstein** cattle have been intensively selected during recent centuries, especially in the last few decades after the implementation of progeny-test-based breeding programs in the 1960s^9,10^. This breed has become highly specialized for milk production. Korean beef producers have selected **Hanwoo** for meat yield and quality^11^. Hanwoo is reported to have originated from crossbreeding between taurine and zebu cattle and migrated to the Korean peninsula through North China; their history as a draft animal dates back at least 5,000 years^12^. **Angus** cattle, (Aberdeen Angus) are beef breed, and they were developed from cattle native to the counties of [Aberdeenshire](https://en.wikipedia.org/wiki/Aberdeenshire_(historic)) and in Scotland^13^. **Jersey** cattle are small breed of dairy cattle, originally bred in the Channel Island of Jersey. This breed is popular for the better quality of milk^14^.

REFERENCE

1. DAGRIS. Domestic Animal Genetic Resources Information System (DAGRIS).

(eds S. Kemp, Y. Mamo, B. Asrat and T. Dessie). International Livestok

Research Institute, Addis Ababa, Ethiopia.

2. Mason, I.L. & Maule, J.P. The indigenous livestock of Eastern and Southern Africa. (1960).

3. Wurzinger, M. *et al.* Comparison of production systems and selection criteria of Ankole cattle by breeders in Burundi, Rwanda,Tanzania and Uganda. *Tropical Animal Health and Production* **38**, 571-581 (2006).

4. Rege, J. The state of African cattle genetic resources I. Classification framework and identification of threatened and extinct breeds. *Animal Genetic Resources Information* **25**, 1-25 (1999).

5. Ndumu, D. *et al.* Genetic and morphological characterisation of the Ankole Longhorn cattle in the African Great Lakes region. *Genetics Selection Evolution* **40**, 467-490 (2008).

6. Murray, M. & Trail, J. Trypanotolerance: genetics, environmental influences and mechanisms. (1982).

7. Mattioli, R.C., Pandey, V.S., Murray, M. & Fitzpatrick, J.L. Immunogenetic influences on tick resistance in African cattle with particular reference to trypanotolerant N’Dama (*Bos taurus*) and trypanosusceptible Gobra zebu (*Bos indicus*) cattle. *Acta Tropica* **75**, 263-277 (2000).

8. Yousif, I. & El-Moula, A. Characterisation of Kenana cattle breed and its production environment. *Animal Genetic Resources Information* **38**, 47-56 (2006).

9. Rege, J. The state of African cattle genetic resources I. Classification framework and identification of threatened and extinct breeds. Animal Genetic Resources Information 25, 1-25 (1999).

10. Skjervold, H. & Langholz, H.J. Factors affecting the optimum structure of AI breeding in dairy cattle. Zeitschrift für Tierzüchtung und Züchtungsbiologie 80, 25-40 (1964).

11. Chung, E. & Kim, W. Association of SNP marker in IGF-I and MYF5 candidate genes with growth traits in Korean cattle. ASIAN AUSTRALASIAN JOURNAL OF ANIMAL SCIENCES 18, 1061 (2005).

12. Lee, C. & Pollak, E. Genetic antagonism between body weight and milk production in beef cattle. Journal of animal science 80, 316-321 (2002).

13. Britannica, E. & King, T. Encyclopaedia Britannica Almanac 2009, (Encyclopædia Britannica, 2009).

14. Consortium, B.H. Genome-wide survey of SNP variation uncovers the genetic structure of cattle breeds. Science 324, 528-532 (2009).

**Table S1.** Publically available whole genome sequencing data used for analyses.

| Breed | Sample size | Bioproject ID |
| --- | --- | --- |
| Holstein | 10 | PRJNA210521 |
| Jersey | 10 | PRJNA318089 |
| Angus | 10 | PRJNA318087 |
| Hanwoo | 23 | PRJNA210523 |

**Table S2**. Summary of sequencing data.

| Sample ID | DNA  Sequenced (bp) | Total Reads | Alignment  Rate (%) | Read Depth | Genome  Coverage (%) | SRA |
| --- | --- | --- | --- | --- | --- | --- |
| Bo_672 | 30,638,401,106 | 303,350,506 | 98.77% | 5.97 | 97.78 | SRS1468768 |
| Bo_2612 | 28,403,655,310 | 281,224,310 | 98.92% | 9.07 | 98.61 | SRS1335347 |
| Bo_37506E | 28,301,084,356 | 280,208,756 | 98.64% | 9.09 | 98.56 | SRS1338703 |
| Bo_392205G | 28,325,968,534 | 280,455,134 | 98.58% | 8.99 | 98.56 | SRS1429391 |
| Bo_587 | 31,211,002,224 | 309,019,824 | 98.90% | 9.98 | 98.66 | SRS1727978 |
| Bow_1304 | 30,304,683,976 | 300,046,376 | 98.77% | 9.51 | 98.61 | SRS1468771 |
| Bo_439 | 28,267,720,016 | 279,878,416 | 98.76% | 8.87 | 98.53 | SRS1346805 |
| Bow_1688 | 31,921,906,076 | 316,058,476 | 98.64% | 9.67 | 98.68 | SRS1468770 |
| Bo_467 | 28,829,275,168 | 285,438,368 | 98.80% | 9.15 | 98.55 | SRS1429491 |
| Bo_563005K | 32,477,775,534 | 321,562,134 | 96.16% | 9.85 | 98.73 | SRS1437860 |
| BT200001 | 30,125,956,396 | 298,276,796 | 98.76% | 9.63 | 98.66 | SRS1338704 |
| BT200015 | 26,850,027,254 | 265,841,854 | 98.91% | 8.81 | 98.60 | SRS1496254 |
| BT200030 | 28,511,065,982 | 282,287,782 | 89.16% | 6.92 | 95.31 | SRS1496257 |
| BT200050 | 31,302,879,904 | 309,929,504 | 98.99% | 9.91 | 98.67 | SRS1496259 |
| BT200073 | 30,310,459,964 | 300,103,564 | 98.89% | 9.82 | 98.73 | SRS1496260 |
| BT200109 | 30,781,515,884 | 304,767,484 | 98.85% | 10.04 | 98.61 | SRS1496261 |
| BT200111 | 30,329,107,796 | 300,288,196 | 99.01% | 9.89 | 98.61 | SRS1496262 |
| BT200122 | 29,454,188,226 | 291,625,626 | 98.96% | 9.47 | 98.64 | SRS1496264 |
| BT200123 | 28,797,582,580 | 285,124,580 | 98.94% | 9.42 | 98.64 | SRS1496265 |
| BT200135 | 29,320,591,284 | 290,302,884 | 98.96% | 9.43 | 98.65 | SRS1496266 |
| KENANA_2 | 30,839,778,340 | 305,344,340 | 98.87% | 9.69 | 98.61 | SRS1439369 |
| KENANA_4 | 31,233,638,748 | 309,243,948 | 98.80% | 9.80 | 98.63 | SRS1446906 |
| KENANA_11 | 31,017,693,678 | 307,105,878 | 98.78% | 9.74 | 98.59 | SRS1437861 |
| KENANA_13 | 30,171,253,078 | 298,725,278 | 98.78% | 9.45 | 98.60 | SRS1439366 |
| KENANA_14 | 30,161,939,666 | 298,633,066 | 98.79% | 9.67 | 98.60 | SRS1439367 |
| KENANA_16 | 29,802,497,432 | 295,074,232 | 98.84% | 9.52 | 98.61 | SRS1439368 |
| KENANA_7 | 30,075,057,244 | 297,772,844 | 98.78% | 9.52 | 98.59 | SRS1446916 |
| KENANA_18 | 30,979,082,186 | 306,723,586 | 98.74% | 9.63 | 98.74 | SRS1439001 |
| KENANA_20 | 30,609,308,258 | 303,062,458 | 98.87% | 9.68 | 98.61 | SRS1439005 |
| ND064 | 30,210,384,316 | 299,112,716 | 98.99% | 9.96 | 98.65 | SRS1512380 |
| ND118 | 28,909,114,052 | 286,228,852 | 99.09% | 9.31 | 98.62 | SRS1512399 |
| ND131 | 27,437,854,324 | 271,661,924 | 98.11% | 8.73 | 98.56 | SRS1512404 |
| ND148 | 30,088,935,654 | 297,910,254 | 99.09% | 9.72 | 98.63 | SRS1512405 |
| ND158 | 30,552,295,374 | 302,497,974 | 99.08% | 9.96 | 98.66 | SRS1512406 |
| ND166 | 30,441,334,552 | 301,399,352 | 99.14% | 10.20 | 98.70 | SRS1512407 |
| ND169 | 30,187,321,774 | 298,884,374 | 99.12% | 9.79 | 98.66 | SRS1512417 |
| ND183 | 29,920,318,376 | 296,240,776 | 98.96% | 9.48 | 98.65 | SRS1512418 |
| ND719 | 31,590,149,558 | 312,773,758 | 99.12% | 10.07 | 98.67 | SRS1512498 |
| ND730 | 30,095,727,500 | 297,977,500 | 99.10% | 9.76 | 98.65 | SRS1512514 |
| OgD5 | 29,246,322,550 | 289,567,550 | 98.60% | 8.92 | 98.54 | SRS1512521 |
| OgD6 | 31,562,494,748 | 312,499,948 | 98.74% | 9.95 | 98.61 | SRS1512522 |
| OgD7 | 29,799,017,376 | 295,039,776 | 98.72% | 9.40 | 98.57 | SRS1512523 |
| OgD1 | 29,461,012,594 | 291,693,194 | 98.68% | 9.28 | 98.58 | SRS1512518 |
| OgD2 | 30,480,606,584 | 301,788,184 | 98.71% | 9.63 | 98.61 | SRS1512519 |
| OgD8 | 31,422,123,736 | 311,110,136 | 98.79% | 9.41 | 98.55 | SRS1512524 |
| OgD4 | 29,744,012,574 | 294,495,174 | 98.54% | 9.14 | 98.54 | SRS1512520 |
| OgS1 | 26,868,959,300 | 266,029,300 | 98.49% | 8.58 | 98.66 | SRS1512525 |
| OgS2 | 31,218,873,356 | 309,097,756 | 98.62% | 9.67 | 98.69 | SRS1512526 |

**Table S3**. Chromosomal distribution and number of SNPs

| Chromosome | Length (bp)* | Numbers of SNPs | Average distance between SNPs |
| --- | --- | --- | --- |
| 1 | 158,337,067 | 2,307,435 | 68 |
| 2 | 137,060,424 | 1,917,262 | 71 |
| 3 | 121,430,405 | 1,662,248 | 73 |
| 4 | 120,829,699 | 1,762,569 | 68 |
| 5 | 121,191,424 | 1,665,070 | 72 |
| 6 | 119,458,736 | 1,740,133 | 68 |
| 7 | 112,638,659 | 1,533,299 | 73 |
| 8 | 113,384,836 | 1,579,856 | 71 |
| 9 | 105,708,250 | 1,492,154 | 70 |
| 10 | 104,305,016 | 1,476,536 | 70 |
| 11 | 107,310,763 | 1,506,893 | 71 |
| 12 | 91,163,125 | 1,423,996 | 64 |
| 13 | 84,240,350 | 1,158,798 | 72 |
| 14 | 84,648,390 | 1,187,482 | 71 |
| 15 | 85,296,676 | 1,291,360 | 66 |
| 16 | 81,724,687 | 1,167,512 | 69 |
| 17 | 75,158,596 | 1,110,369 | 67 |
| 18 | 66,004,023 | 907,819 | 72 |
| 19 | 64,057,457 | 875,206 | 73 |
| 20 | 72,042,655 | 1,068,977 | 67 |
| 21 | 71,599,096 | 1,031,652 | 69 |
| 22 | 61,435,874 | 863,499 | 71 |
| 23 | 52,530,062 | 864,636 | 60 |
| 24 | 62,714,930 | 948,245 | 66 |
| 25 | 42,904,170 | 627,563 | 68 |
| 26 | 51,681,464 | 767,090 | 67 |
| 27 | 45,407,902 | 719,364 | 63 |
| 28 | 46,312,546 | 742,945 | 62 |
| 29 | 51,505,224 | 819,853 | 62 |
| MT | 16,338 | 319 | 51 |
| X | 148,823,899 | 1,143,296 | 130 |

*Based on UMD3.1 genome of reference

**Table S4.** Genotype concordance rates of African cattle samples

| Sample ID | Loci in  common | Concordant loci  by genotype | Genotype  concordance |
| --- | --- | --- | --- |
| Bo_2612 | 36,403 | 34,656 | 95.2% |
| Bo_37506E | 36,321 | 34,461 | 94.9% |
| Bo_392205G | 36,153 | 34,344 | 95.0% |
| Bo_439 | 30,075 | 28,268 | 94.0% |
| Bo_467 | 26,829 | 25,200 | 93.9% |
| Bo_672 | 33,544 | 31,553 | 94.1% |
| Bow_1304 | 30,109 | 28,597 | 95.0% |
| Bow_1688 | 35,556 | 33,858 | 95.2% |
| BT200001 | 34,091 | 31,413 | 92.1% |
| BT200015 | 36,590 | 34,834 | 95.2% |
| BT200030 | 36,802 | 32,869 | 89.3% |
| BT200050 | 36,215 | 34,600 | 95.5% |
| BT200073 | 33,673 | 29,075 | 86.3% |
| BT200109 | 30,323 | 28,911 | 95.3% |
| BT200111 | 36,084 | 34,508 | 95.6% |
| BT200122 | 36,899 | 35,252 | 95.5% |
| BT200123 | 36,647 | 34,229 | 93.4% |
| BT200135 | 35,651 | 32,120 | 90.1% |
| KENANA_11 | 40,392 | 38,872 | 96.2% |
| KENANA_13 | 40,001 | 38,463 | 96.2% |
| KENANA_14 | 35,577 | 34,148 | 96.0% |
| KENANA_16 | 35,154 | 33,746 | 96.0% |
| KENANA_18 | 33,527 | 31,940 | 95.3% |
| KENANA_2 | 34,716 | 33,289 | 95.9% |
| KENANA_20 | 37,535 | 35,941 | 95.8% |
| KENANA_4 | 39,093 | 37,601 | 96.2% |
| ND064 | 37,028 | 35,506 | 95.9% |
| ND118 | 36,904 | 35,210 | 95.4% |
| ND131 | 36,327 | 34,249 | 94.3% |
| ND148 | 32,033 | 30,241 | 94.4% |
| ND158 | 29,075 | 27,532 | 94.7% |
| ND166 | 39,324 | 37,798 | 96.1% |
| ND169 | 41,238 | 39,432 | 95.6% |
| ND183 | 40,885 | 39,209 | 95.9% |
| ND719 | 38,286 | 36,923 | 96.4% |
| ND730 | 38,164 | 36,812 | 96.5% |
| OgD1 | 39,454 | 37,828 | 95.9% |
| OgD2 | 40,108 | 38,557 | 96.1% |
| OgD4 | 36,741 | 35,075 | 95.5% |
| OgD5 | 34,983 | 33,428 | 95.6% |
| OgD6 | 35,423 | 33,944 | 95.8% |
| OgD7 | 38,686 | 37,104 | 95.9% |
| OgD8 | 39,525 | 37,982 | 96.1% |
| OgS1 | 39,689 | 38,051 | 95.9% |
| OgS2 | 36,827 | 35,420 | 96.2% |

**Table S5.** Number of significant (Bonferroni adjusted *P*-value < 0.05) MISSENSE and NONSENSE (stop gained) variants specific to each breed

| Breed | MISSENSE | | | NONSENSE | | | The others | | |
| --- | --- | --- | --- | --- | --- | --- | --- | --- | --- |
| Angus | 24 | 2 | 333 | 1 | 0 | 2 | 6,305 | 734 | 84,765 |
| Ankole | 14 | 11 | 252 | 0 | 0 | 1 | 4,323 | 634 | 87,405 |
| Boran | 6 | 2 | 351 | 0 | 0 | 0 | 3,148 | 713 | 173,985 |
| Hanwoo | 348 | 45 | 537 | 1 | 0 | 1 | 119,495 | 18,056 | 175,435 |
| Holstein | 62 | 21 | 592 | 0 | 0 | 1 | 17,827 | 8,389 | 167,574 |
| Jersey | 175 | 130 | 1,103 | 1 | 0 | 3 | 47,758 | 31,394 | 316,721 |
| Kenana | 0 | 0 | 177 | 0 | 0 | 1 | 304 | 41 | 97,544 |
| N’Dama | 138 | 62 | 1,009 | 1 | 0 | 2 | 37,040 | 23,682 | 313,382 |
| Ogaden | 0 | 0 | 114 | 0 | 0 | 1 | 110 | 29 | 60,362 |
| Genetic model | DOM | REC | Geno | DOM | REC | Geno | DOM | REC | Geno |

**Table S6.** *F_ST_* estimates between groups of breeds used for comparative analyses.

|  | African  *vs*  Commercial | N’Dama  *vs*  African | Ankole  *vs*  African | Boran  *vs*  African | Kenana  *vs*  African | Ogaden  *vs*  African | Indicus  *vs*  Commercial |
| --- | --- | --- | --- | --- | --- | --- | --- |
| F_ST_ | 0.200 | 0.172 | 0.044 | 0.053 | 0.039 | 0.039 | 0.292 |

**Table S7.** Summary of non-synonymous polymorphisms detected within HCRTR1 gene region on BTA2.

| Variant Position | Base change | Amino acid change |
| --- | --- | --- |
| 122642051 | cGg/cAg | R405Q |
| 122645430 | cGg/cAg | R260Q |
| 122645433 | aAg/aGg | K259R |
| 122647588 | Cgg/Tgg | R197W |
| 122647614 | gTg/gCg | V188A |
| 122647693 | Cgg/Tgg | R162W |
| 122649547 | Gta/Ata | V59I |

**Figure S1.** Principal Component Analysis of African cattle breeds (a), commercial breeds (b), and taurine breeds (c).


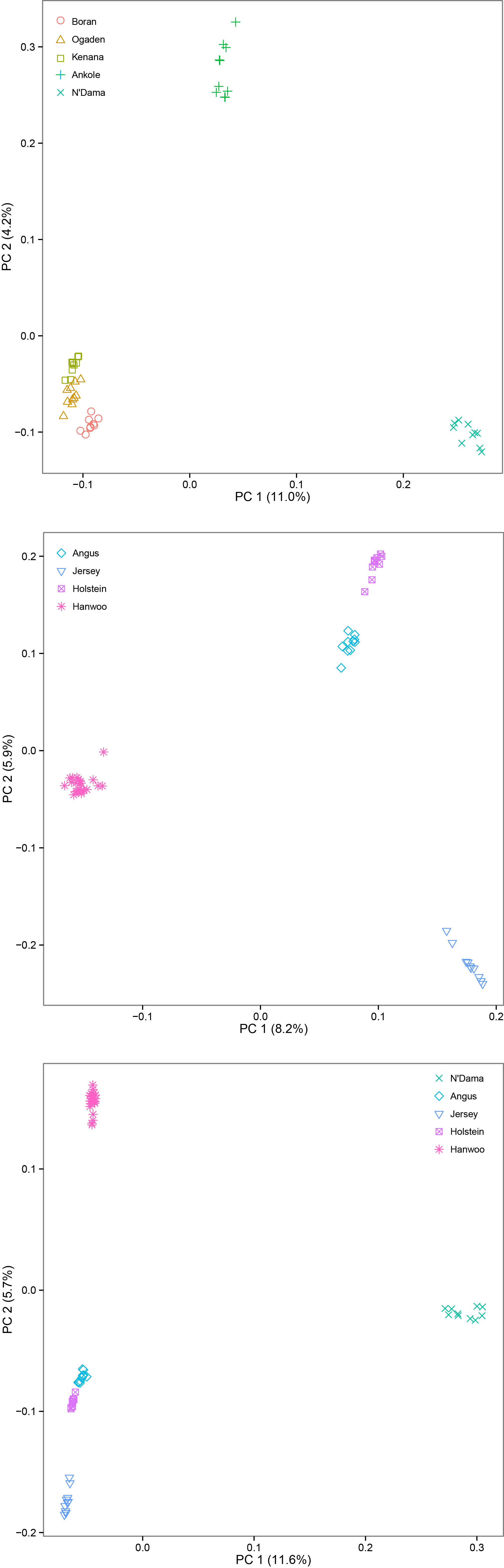


**Figure S2.** Proportion of ancestry for each individual assuming different numbers of ancestral population (K = 6 and 9). Colors in each vertical line represent the likelihood proportion of an animal genome assigned to a source population. Plot of delta (K) model scores using the Evanno method (bottom) indicating that K=2 has the highest delta K and therefore represents the best fit to the data.

**
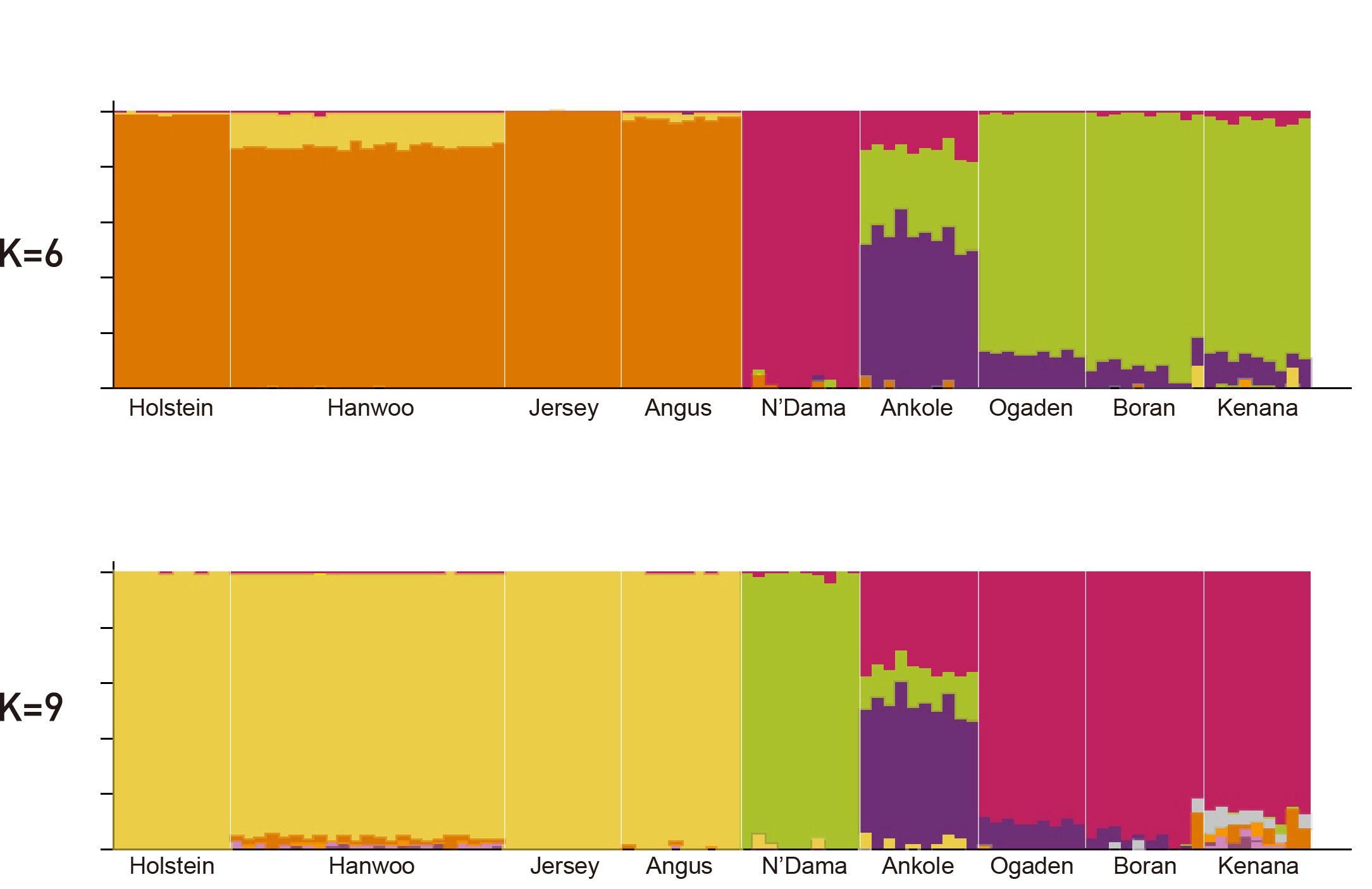
**

**
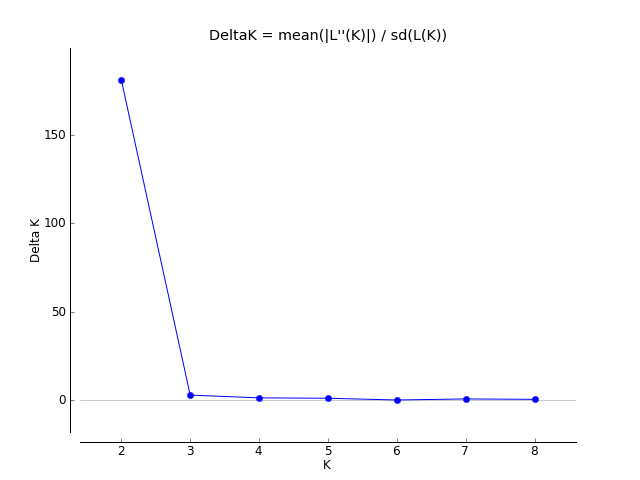
**

### Figure S3. 90% credible intervals of estimated population size history in each cattle breed.

###
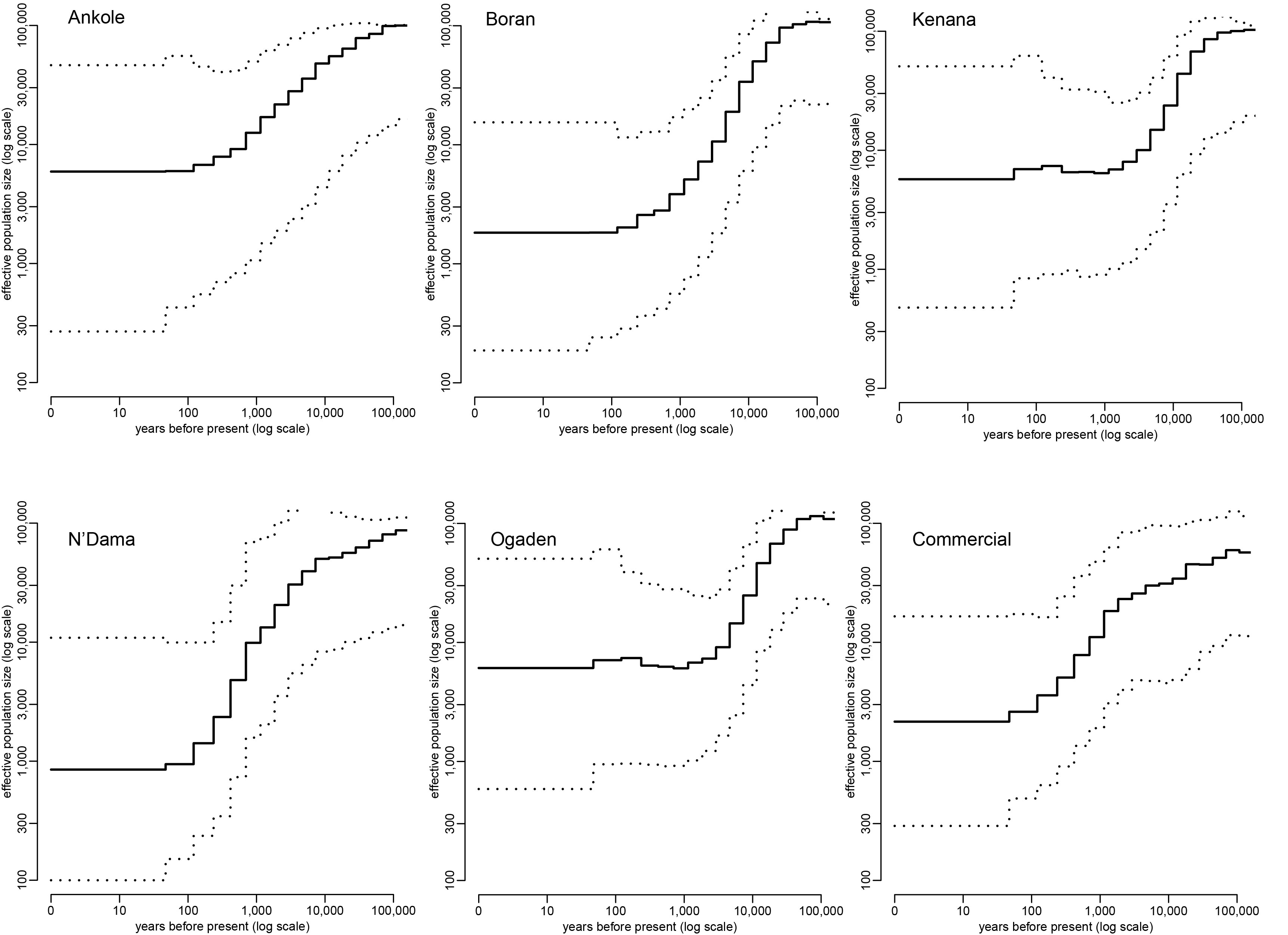


**Figure S4.** The maximum likelihood tree of the nine breeds and the residual matrix of the maximum likelihood tree assuming one and two migration events using *TreeMix*. The scale bar represents ten times the average standard error (s.e.) of the values in the covariance matrix. Positive residuals indicate pairs of breeds where the fit might be improved by adding additional edges.


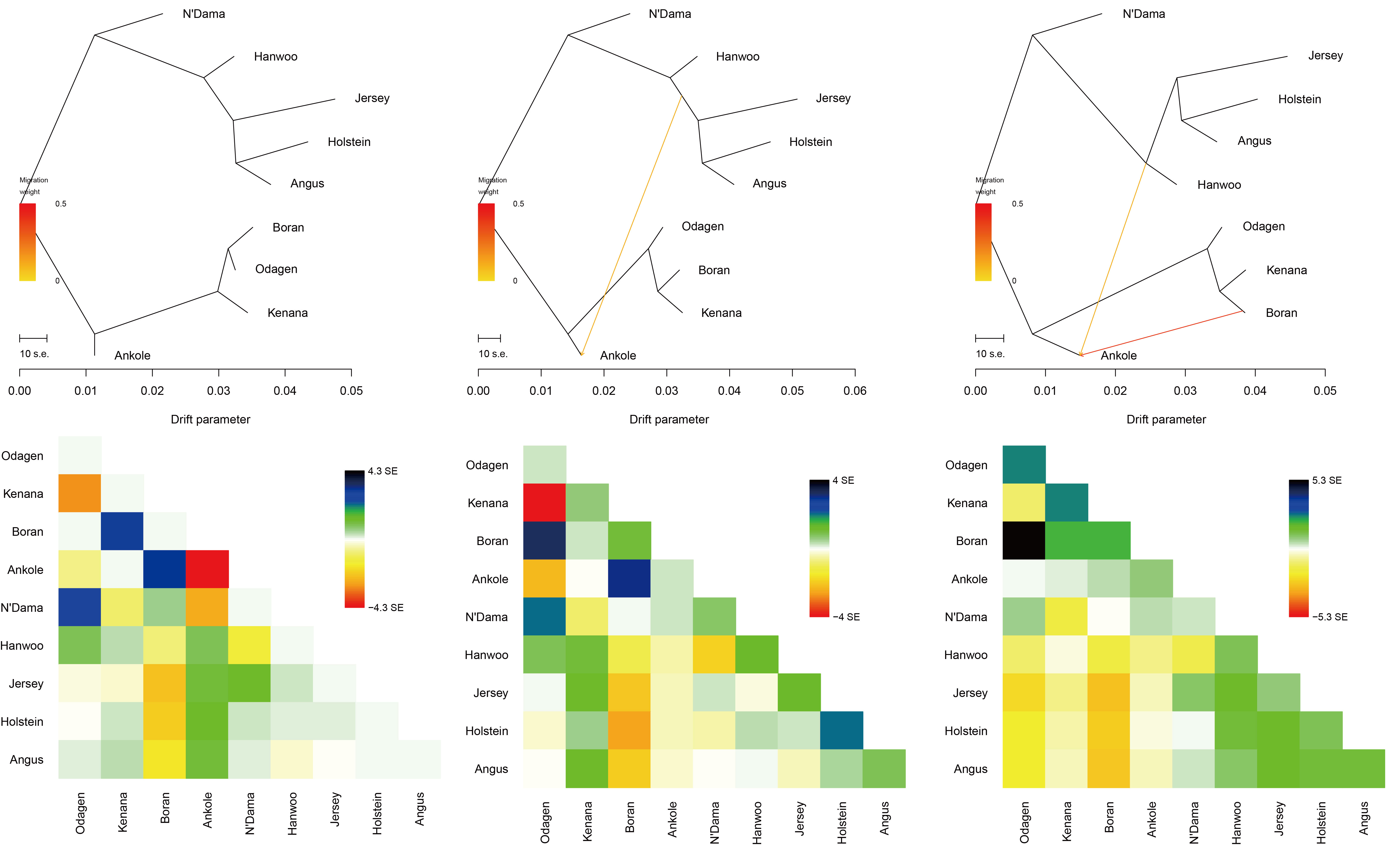


**Figure S5**. Distribution plots of XP-EHH raw score for each population comparison.


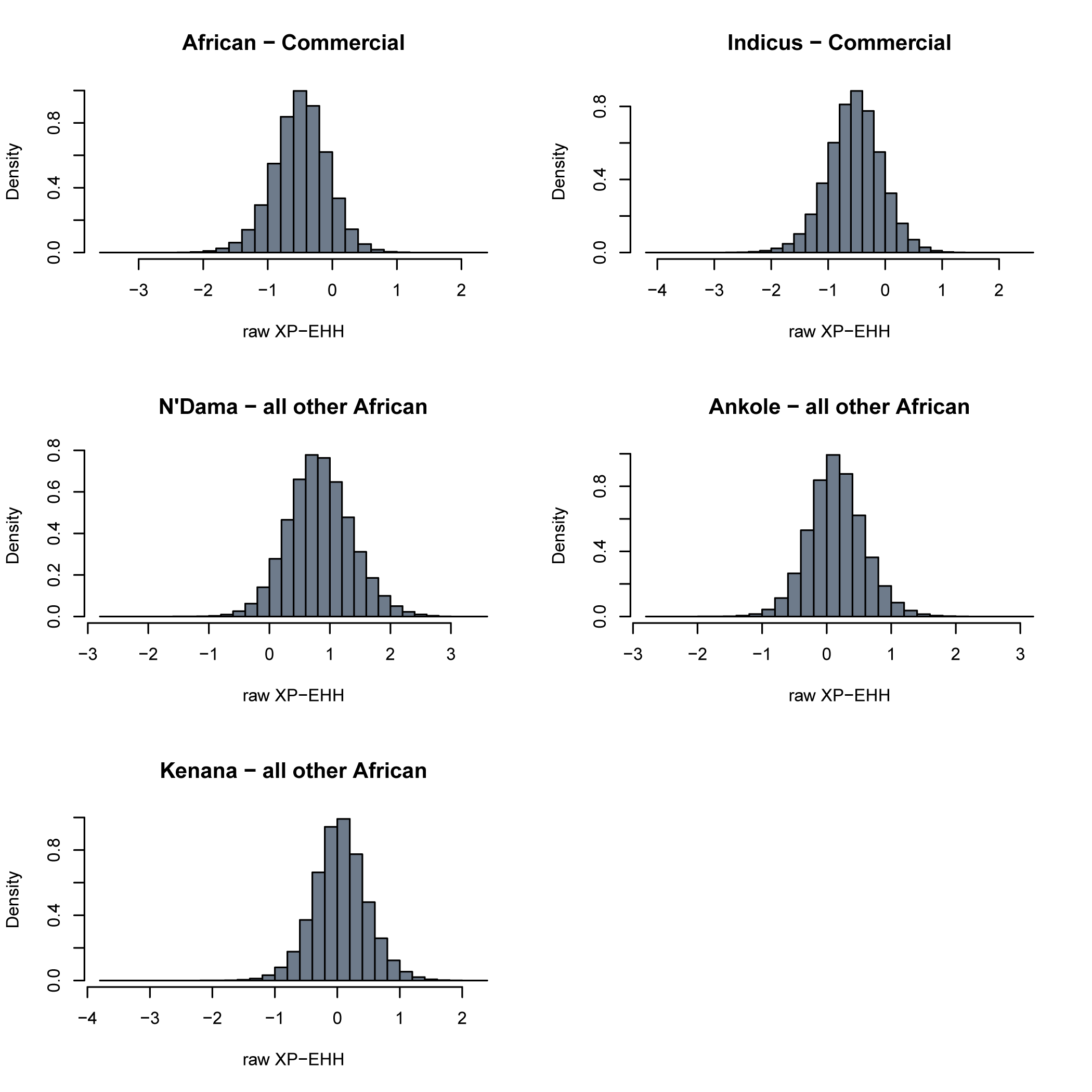


**Figure S6**. Histogram of SNP density in non-overlapping 50 kb window


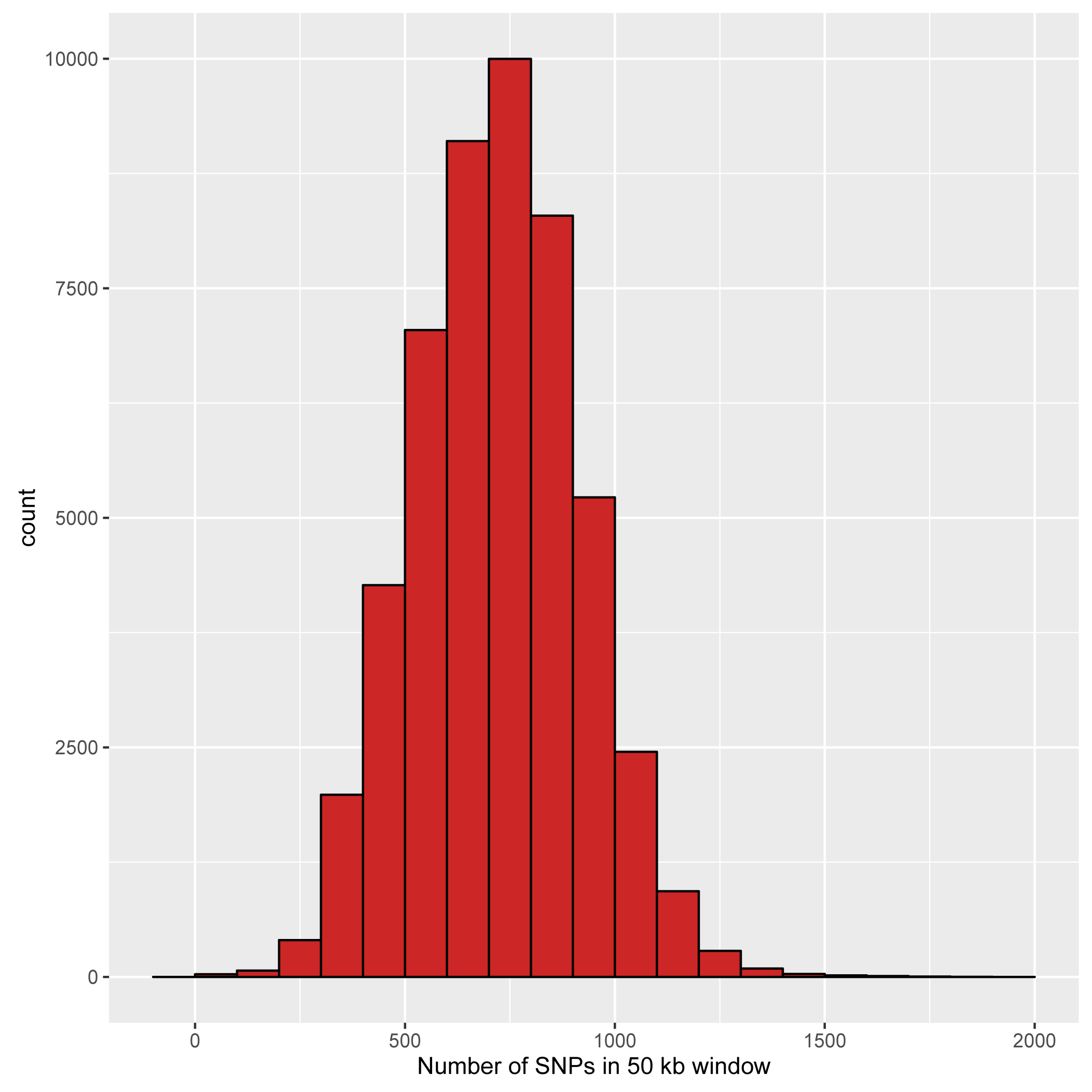


**Figure S7.** Distribution plots of XP-CLR raw score for each population comparison.


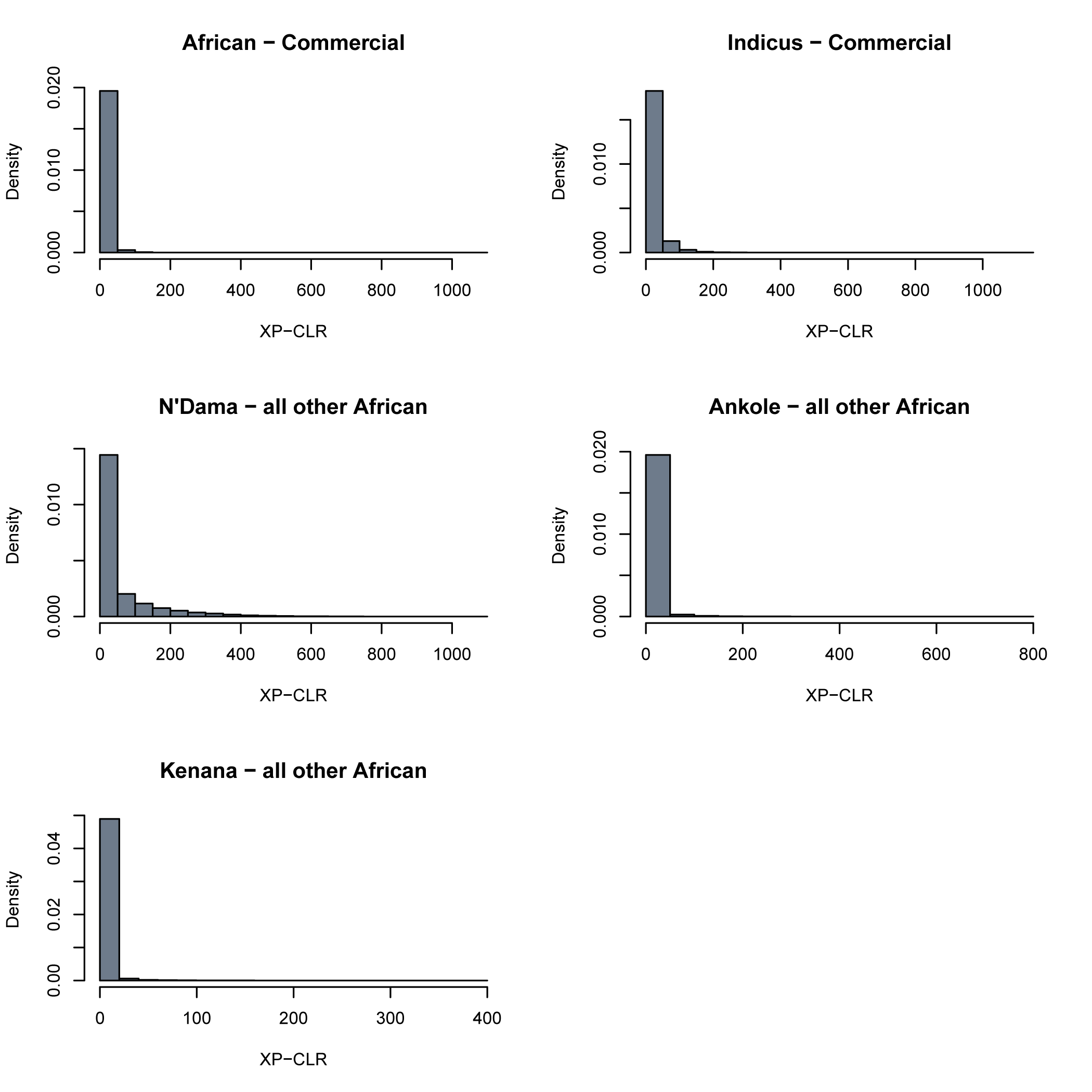


**Figure S8**. Haplotype sharing plot at *MC1R* and *KIT* gene regions. Numbers at the bottom represent genome coordinates.


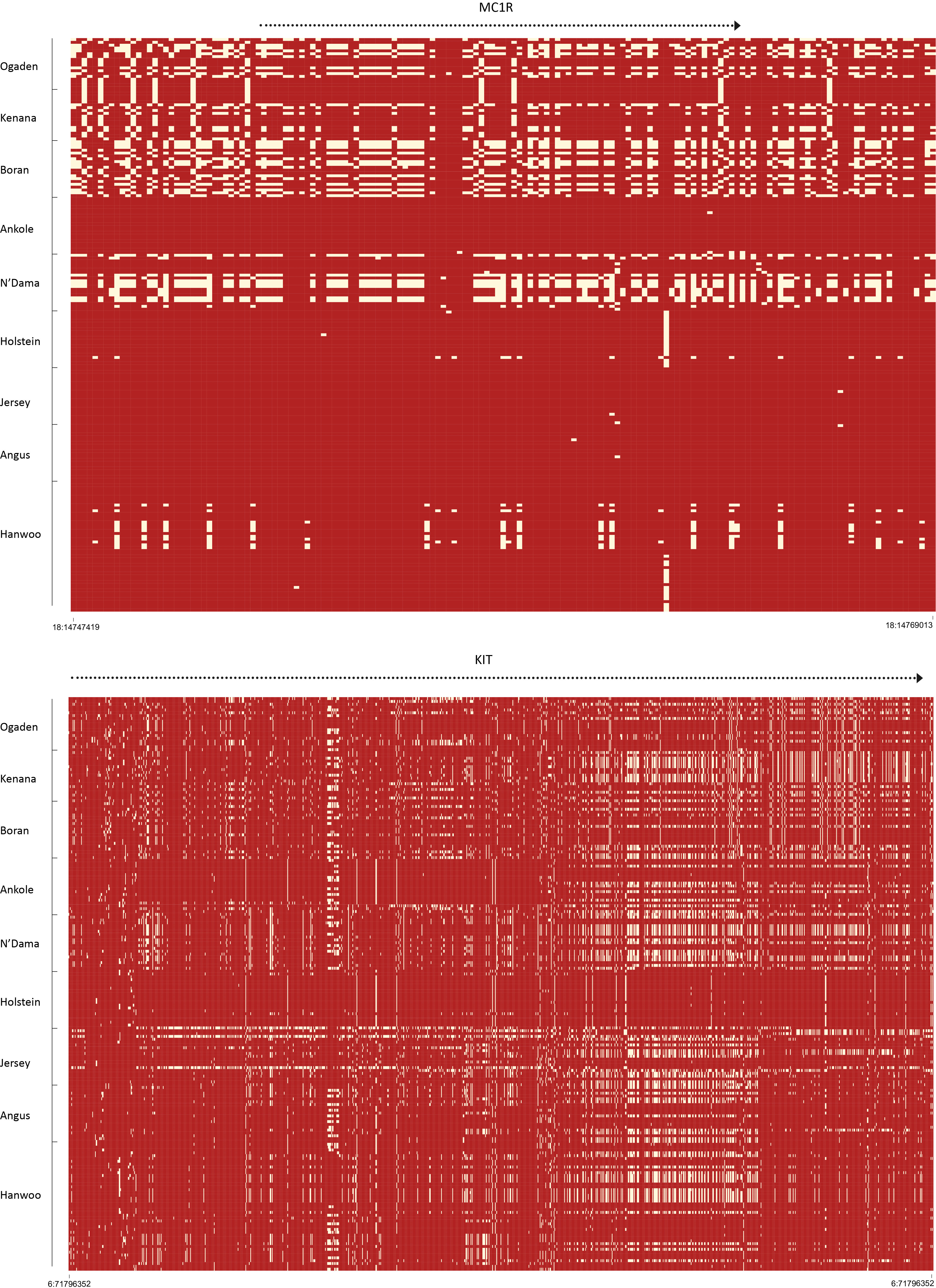


Figure S9. Haplotype blocks frequencies at the BoLA gene. *denotes non-synonymous SNPs.


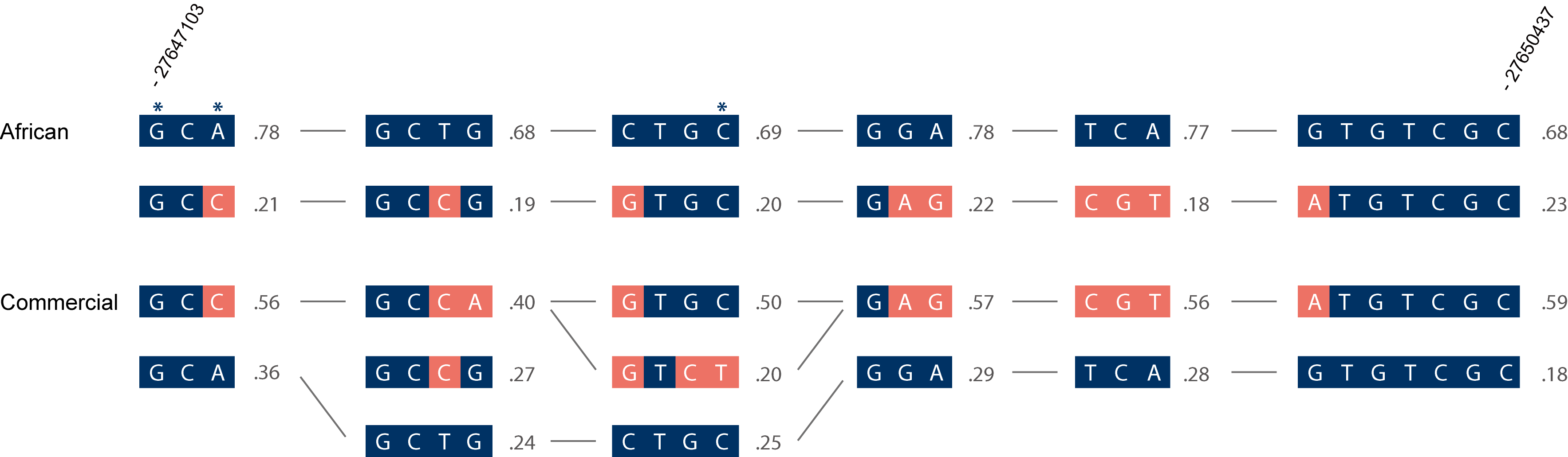


**Figure S10.** Pattern of haplotype sharing in random genomic region of 1Mb. The major allele in each *Bos taurus* and *Bos indicus* cattle is indicated in orange.


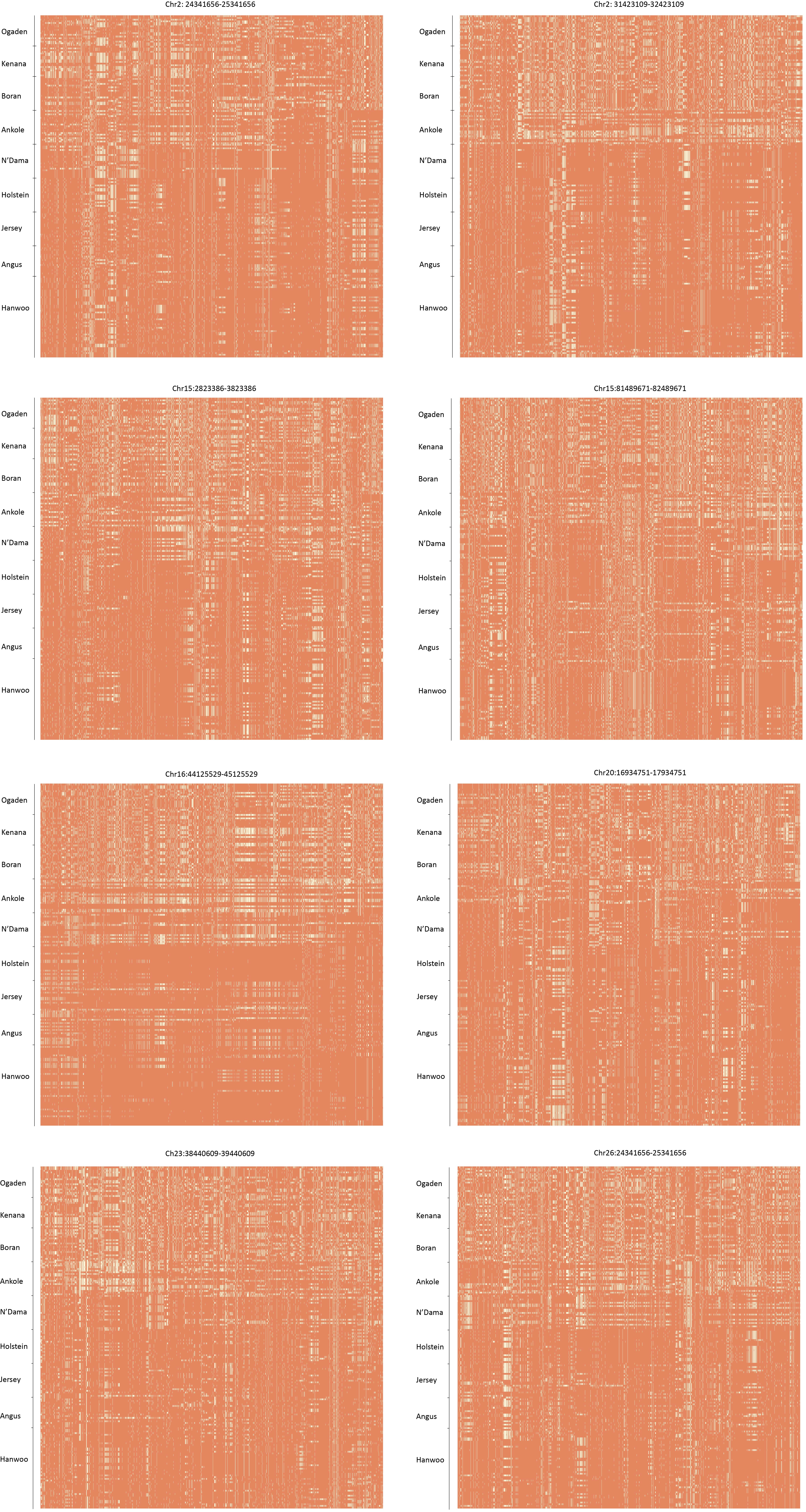


**Figure S11**. Haplotype sharing plot of HSPA4 gene region. Numbers at the bottom represent genome coordinates.


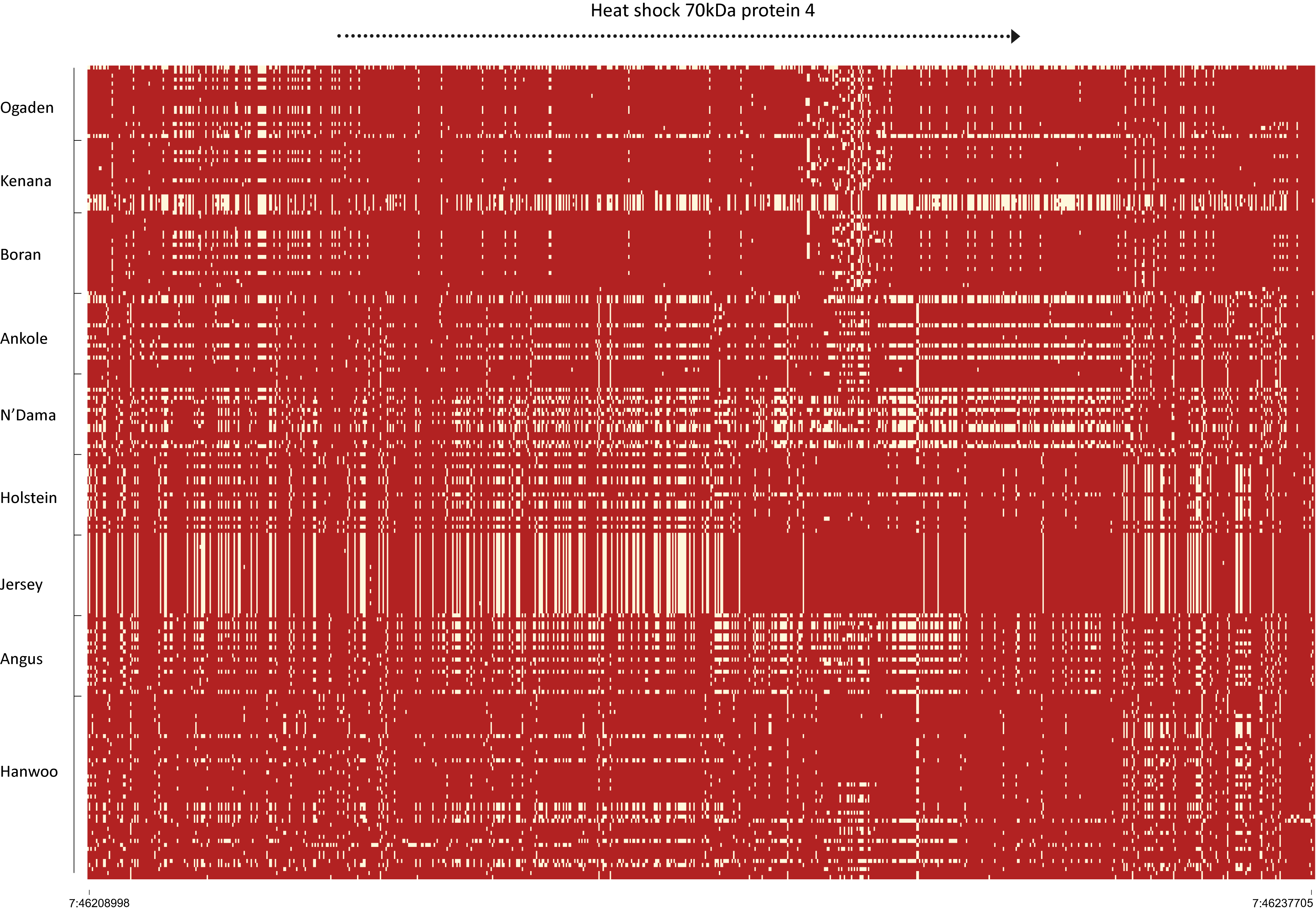


**Figure S12.** Structure of the *PRLH* gene with exons indicated by vertical bars. A non-synonymous SNP represents p.Arg76His and is highlighted in yellow. Haplotype frequencies are indicated by numbers next to each haplotype. In each haplotype, green and beige bars represent allele 1 and 2, respectively.


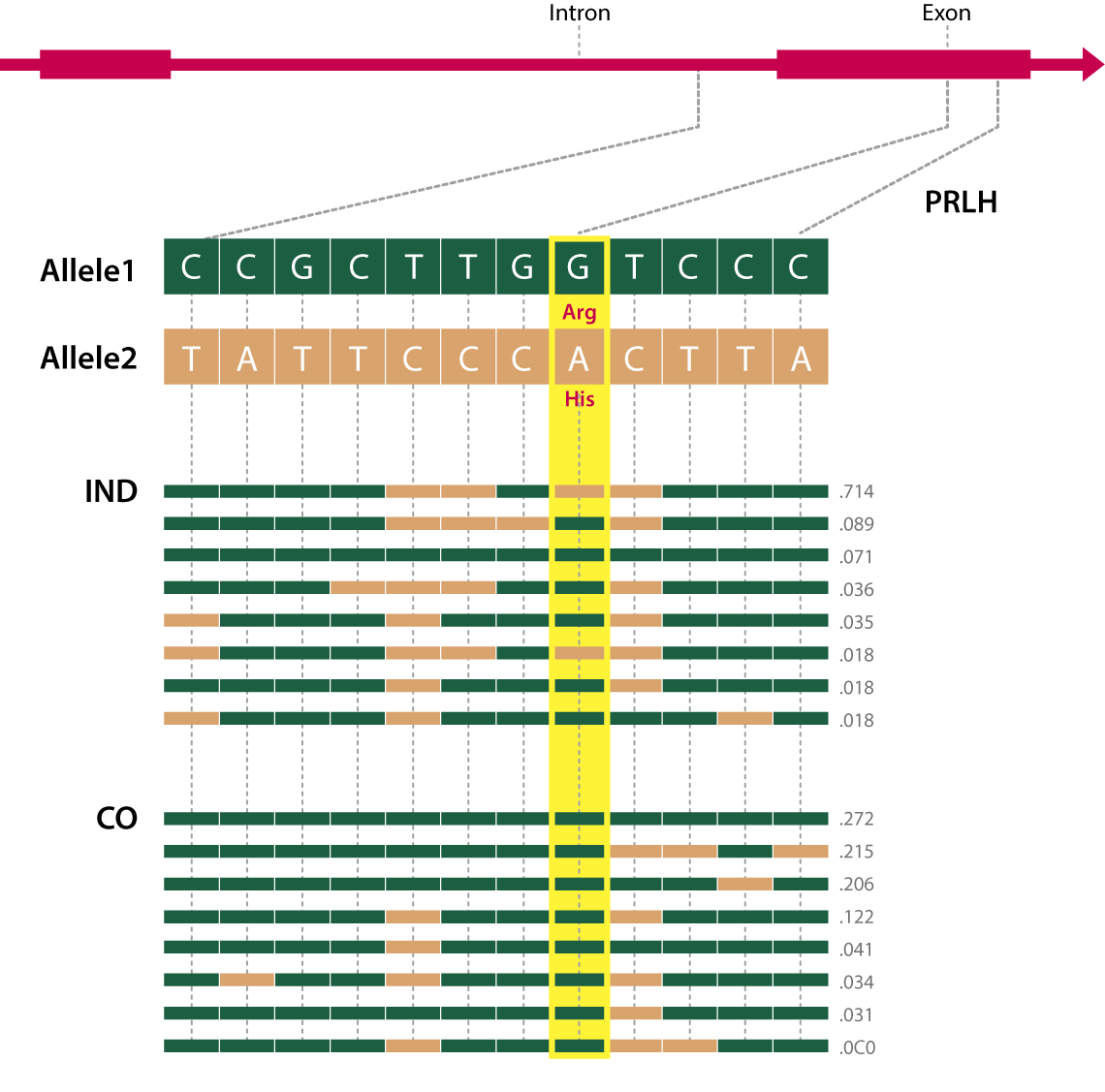

Supplement: Additional file 1: — Supplemental data including Note S1, Tables S1-S7 and Figures S1-S12. (DOCX 14235 kb) [file 13059_2017_1153_MOESM1_ESM.docx]
